# Development of an In Vitro Biomimetic Peripheral Neurovascular Platform

**DOI:** 10.1021/acsami.2c03861

**Published:** 2022-07-11

**Authors:** Afonso Malheiro, Adrián Seijas-Gamardo, Abhishek Harichandan, Carlos Mota, Paul Wieringa, Lorenzo Moroni

**Affiliations:** Complex Tissue Regeneration Department, MERLN Institute for Technology-Inspired Regenerative Medicine, Maastricht University, 6229 ET Maastricht, The Netherlands

**Keywords:** neurons, endothelial cells, Schwann cells, neurovascular interactions, in vitro model, three-dimensional

## Abstract

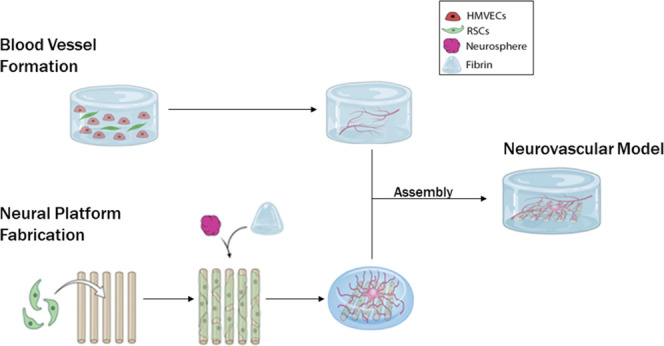

Nerves and blood vessels are present in most organs and are indispensable for their function and homeostasis. Within these organs, neurovascular (NV) tissue forms congruent patterns and establishes vital interactions. Several human pathologies, including diabetes type II, produce NV disruptions with serious consequences that are complicated to study using animal models. Complex in vitro organ platforms, with neural and vascular supply, allow the investigation of such interactions, whether in a normal or pathological context, in an affordable, simple, and direct manner. To date, a few in vitro models contain NV tissue, and most strategies report models with nonbiomimetic representations of the native environment. To this end, we have established here an NV platform that contains mature vasculature and neural tissue, composed of human microvascular endothelial cells (HMVECs), induced pluripotent stem cell (iPSCs)-derived sensory neurons, and primary rat Schwann cells (SCs) within a fibrin-embedded polymeric scaffold. First, we show that SCs can induce the formation of and stabilize vascular networks to the same degree as the traditional and more thoroughly studied human dermal fibroblasts (HDFs). We also show that through SC prepatterning, we are able to control vessel orientation. Using our NV platform, we demonstrate the concomitant formation of three-dimensional neural and vascular tissue, and the influence of different medium formulations and cell types on the NV tissue outcome. Finally, we propose a protocol to form mature NV tissue, via the integration of independent neural and vascular constituents. The platform described here provides a versatile and advanced model for in vitro research of the NV axis.

## Introduction

Nerves and blood vessels (BVs) are commonly found in the same regions, forming overlapping arborized networks/patterns within tissues.^1^ This alignment is the result of intimately linked developmental pathways of these two systems, with each navigating side by side as tissues grow. This neurovascular (NV) alignment evolves into codependency as tissues mature since large nerves require vascularization to ensure nutrient and oxygen supply, whereas large BVs rely on innervation to regulate vasodilation and vasoconstriction.^2−4^ The underlying reason for this shared organization and similar distribution of nerves and BVs is to provide sufficient coverage of a target tissue to ensure survival and function. Parallels also exist in terms of how these target tissues become innervated and vascularized; for both subunits of the NV axis, a tissue releases growth factors (GFs), in soluble or matrix-bound form, to attract and direct nerve and BV development, survival, and growth.^1^ Numerous GFs and receptors are common to both nerve and vessel networks.^5,6^ Nerve growth factor (NGF), for instance, is a known neurotrophic factor but can also exert a positive influence on endothelial cell (EC) proliferation, survival, and migration. Similarly, vascular endothelial growth factor (VEGF) is known to induce vasculogenesis and angiogenesis but can also promote neurogenesis.^5^ In both cases, once the target tissue is stimulated by neuropeptides or sufficiently supplied with oxygen, the production of GFs by the target tissue subsides.^1,7^

Besides common molecular players, there is also mounting evidence of direct influence of nerves on vessels, and vice versa, which results in the stereotyped NV alignment. For example, smooth muscle cells lining the vessels secrete artemin to induce sympathetic nerve fiber alignment.^8^ Conversely, within the skin, Schwann cells (SCs) associated with sensory nerves instruct vessel patterning via local VEGF secretion.^9^ SC and EC interactions, in particular, have been more thoroughly investigated, with in vitro reports showing a promotion of EC migration by SCs^10^ and in vivo reports showing that BVs direct the migration of SC cords during nerve regeneration.^11^ However, there is scarce evidence regarding the vasculogenic/angiogenic potential of SCs.

Given the tight interrelationship between nerves and BVs, it is not surprising that disruptions in one tissue provoke dysfunction in the other. For instance, in diabetes, a worldwide prevalent disease, microvascular damage is frequent, which in turn affects the function of peripheral nerves (PN). Alterations to the microvasculature lead to hypoxia that causes increased oxidative stress, inflammation, and loss of trophic support for neurons and SCs.^12^ Thus, a better understanding of the NV axis would provide important knowledge about native NV communication and give insight for appropriate interventions to combat dysfunctions. To this matter, in vitro NV models can offer a reproducible research platform to investigate pathologies, conduct drug screenings, and decode complex NV interactions in a simple and convenient manner. Compared to animal models, human-based in vitro models offer more clinically relevant data, are more cost-effective, and permit concise and focused investigations on relevant tissues. Additionally, the assembly of peripheral neurovasculature within tissue-engineered (TE) constructs is necessary for the realization of fully functional and mature in vitro organ models.

To date, most in vitro NV models replicate the central nervous system NV unit.^13−15^ Peripheral nerve (PN) NV models include the work of Grasman et al.,^16^ in which human umbilical vein ECs (HUVECs) stimulated axonal growth from a rat dorsal root ganglion population (DRG). However, this model has some drawbacks: first, HUVECs originate from a noninnervated tissue, the umbilical cord, thus limiting the model relevance; second, DRG extraction requires recurrent animal sacrifice, which poses ethical concerns and is an expensive procedure; finally, the co-cultures were established on a glass coverslip, which does not provide the three-dimensional (3D) support required for the proper development and maturation of both tissues. Yuan et al.^17^ described a similar system but containing human microvascular endothelial cells (HMVECs) as a vascular population instead. These cells originate from the skin, a richly innervated tissue, and thus constitute a more relevant cell source. The authors discovered that co-cultures of HMVECs and DRGs led to higher overall cell viability and higher expression of VEGF and NGF, compared to single cultures of each cell population. Yet, the use of a flat substrate in this culture system limits again the model biomimicry, particularly the vascular tissue, which cannot form lumenized capillaries on a two-dimensional (2D) space. To improve upon this, Osaki et al.^18^ proposed an approach that uses embryonic stem cell-derived motor neurons (ESC-MNs) and induced pluripotent stem cell (iPSC)-derived ECs cultured on a collagen gel, to form a 3D NV unit within a microfluidic chip. The authors found that the presence of ECs improved neurite length and function. Despite the shown technological advances, the depicted vascular networks do not present a mature vessel morphology and phenotype. Moreover, the absence of SCs excludes the possibility of forming myelinated axons and oversimplifies the model of an NV milieu, where the presence of different cell types is crucial for proper tissue function.

Here, we report the formation of a 3D NV platform, composed of human iPSC-derived sensory (nociceptor) neurons, HMVECs, and SCs. First, we describe the potential of SCs to induce vasculogenesis of HMVECs in a fibrin hydrogel, with a similar outcome to the well-established human dermal fibroblasts (HDFs). We further conclude that SC-conditioned medium, despite enhancing tubule formation on Matrigel-coated surfaces, is not sufficient to induce 3D vessel formation in fibrin hydrogels. We also demonstrate that, by prepatterning of SCs with an aligned microfibrous scaffold, we are able to direct vessel orientation. To fabricate an NV platform, we used a previously developed neural model^19^ composed of a 3D co-culture of functional human iPSCs-derived sensory neurons and SCs, seeded on a microfibrous scaffold and embedded in a fibrin hydrogel. As a result, we were able to obtain a vast anisotropic and myelinated neurite network. From this, we explored different strategies to include a vascular component and create a peripheral NV unit. Finally, we show that through initial segregation of neural and vascular cultures, followed by integration into a single unit, we are able to generate a mature NV model that shows hallmarks of the native NV interactions, such as NV alignment. We believe that the proposed model is a significant advancement in the biofabrication of a complex, multicellular peripheral NV unit. The platform presented here can be utilized to study and manipulate NV interactions and NV-associated pathologies in a simple, precise, and affordable manner. Furthermore, the TE principles here described can be valuable to overcome challenges in the development of multi-tissue organ platforms containing a peripheral NV unit.

## Results

### Formation of a 3D PN Platform

**Figure 1:**
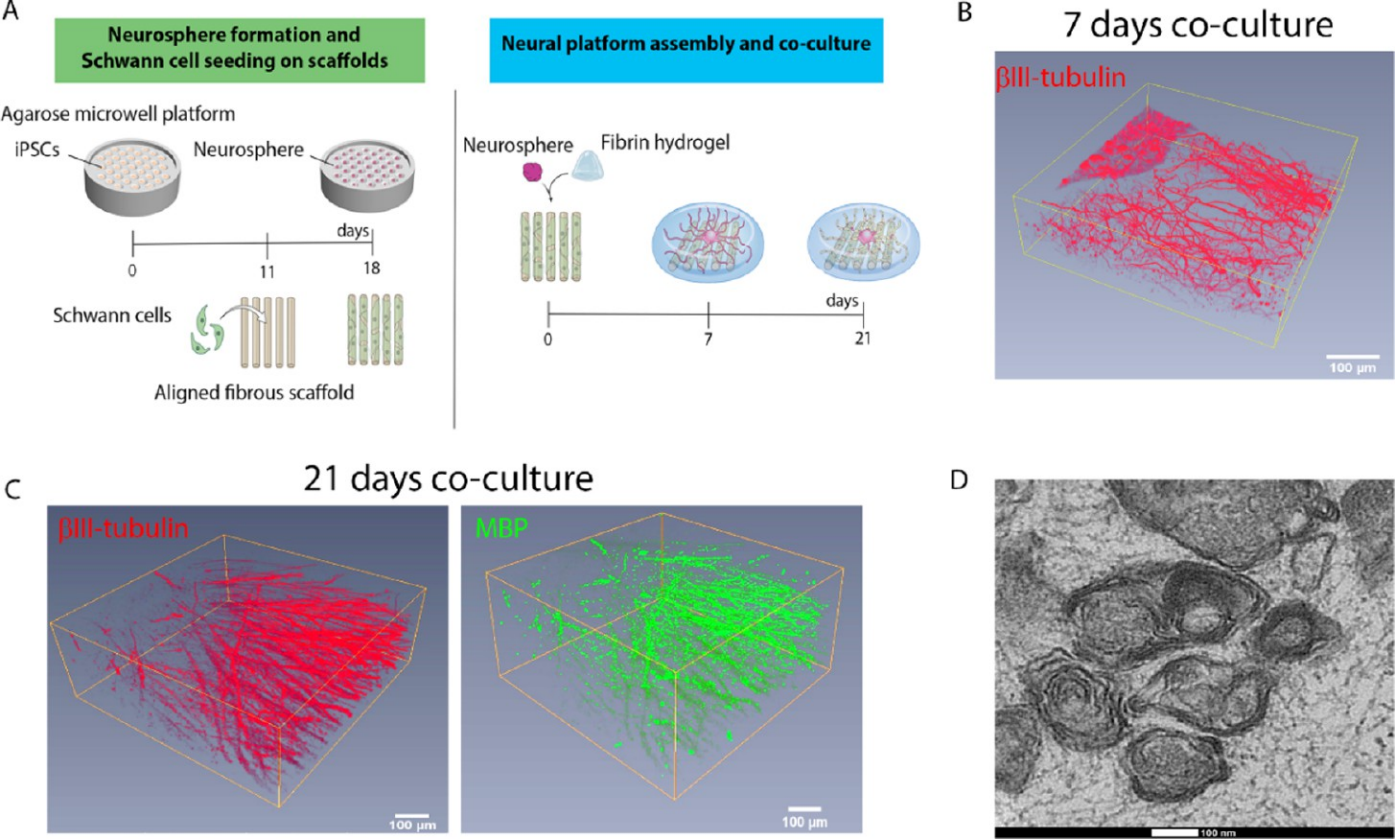
Development of a 3D biomimetic PN platform. (A) Illustration of the biofabrication process, depicting its two main phases. The first phase (left) is the formation of nociceptor neurospheres *via* iPSCs differentiation within an agarose mold containing 400 μm microwells. The whole process takes 18 days from the day cells are seeded (at a density of 200 cells per well) until the spheres are ready to be harvested. The differentiation process itself takes 14 days. On day 11, primary SCs are seeded and cultured on an aligned microfibrous scaffold (at a density of 100 × 10^3^ cells per scaffold). In the second phase (right), the neurospheres are harvested and placed on the SC-seeded scaffold (one neurosphere per scaffold). A fibrin hydrogel embedding is also added, following neurosphere attachment. The co-culture is maintained for up to 21 days to allow compact myelination to occur. (B) At 7 days of co-culture, vast, 3D, and aligned neurite outgrowth was observed, illustrated by the 3D reconstruction of a micrograph showing immunostaining to βIII-tubulin (red). (C) At 21 days of co-culture, neurites maintain their anisotropy and show signals of myelination as evidenced by the 3D reconstruction of a micrograph, correlating βIII-tubulin^+^ (red) with MBP^+^ segments (green). For (C) and (D), the scale bars represent 100 μm. (D) TEM micrograph of the platform cross section showing the presence of multiple, compact myelin rings surrounding the neurites (average thickness is 89.1 ± 17.6 nm from 11 measurements). The scale bar represents 100 nm.

To create an NV unit, we first sought out to develop a platform that would allow neural tissue to grow in a manner that replicates the native PN environment. For this, we used iPSC-derived neurons, in the form of spheroids (neurospheres), and primary SCs, co-cultured on an aligned microfibrous scaffold and embedded wtihin fibrin (Figure 1A). The hydrogel provides the 3D support necessary for cells to grow beyond the 2D scaffold substrate. Additionally, it allows the inclusion of other tissues, in this case, vascular tissue. The co-cultures were maintained up to 21 days to allow proper tissue maturation, i.e., axon myelination. The first checkpoint was on day 7 of culture, where we could see that neurite growth in 3D was already vast and well aligned (Figure 1B). At 21 days, we observed an increase in the neurite volume and the formation of surrounding myelin (Figure 1C). When analyzing the construct cross section via transmission electron microscopy (TEM), we observed the presence of compact and abundant myelin layers, with an average thickness of 89.1 ± 17.6 nm (Figure 1D).

### Formation of a 3D Vascular Platform with HMVECs and HDFs

**Figure 2:**
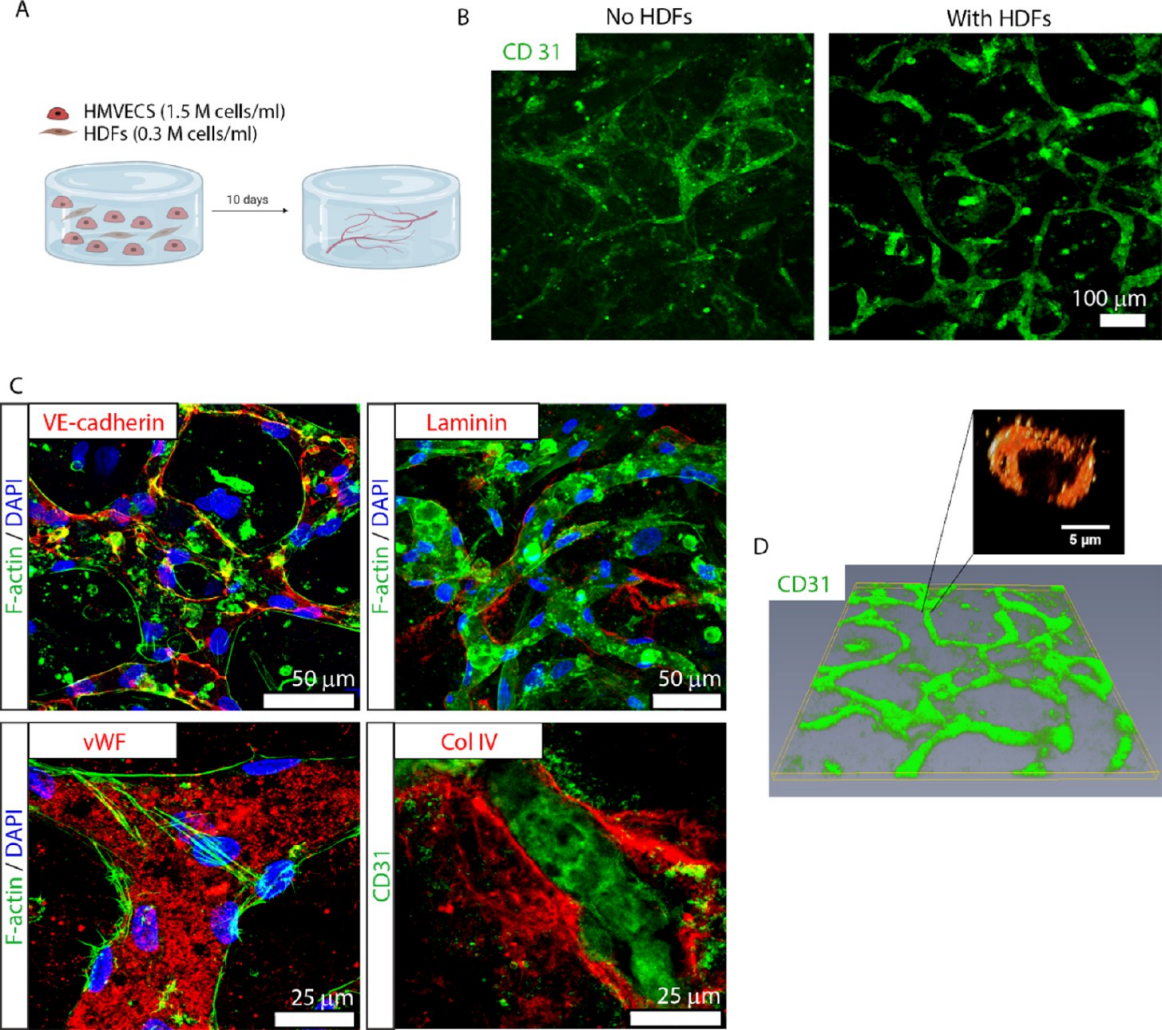
Formation and characterization of a 3D vascular channel network composed of HMVECs and HDFs. (A) Illustration of the biofabrication process. Each 3D culture is composed of 300 μL of human fibrin containing HMVECs, at 1.5 M cells/mL and HDFs, at 0.3 M cells/mL, forming a ratio of 5:1. The culture is maintained for at least 10 DIV. Illustration made with biorender (https://biorender.com/). (B) Presence of HDFs within the culture is essential for the formation and maintenance of well-defined vessels. The micrograph on the left-hand side shows poor vessel formation in the absence of HDFs. In comparison, the presence of HDFs promotes the development of well-defined and interconnected vascular channels (right micrograph). The vascular channel formation is denoted by CD31 immunostaining (green). Scale bar represents 100 μm. (C) Characterization of the vessels’ phenotype with the traditional vascular markers (red in each respective panel): VE-cadherin (top left), laminin (top right), von Willebrand factor (vWF; bottom left), collagen type IV (col IV; bottom right), and CD31 (green, bottom right). F-actin for cytoskeleton (green) and 4′,6-diamidino-2-phenylindole (DAPI) for nuclei (blue) (top row and bottom left) are also shown. The scale bars represent 50 μm (top row) and 25 μm (bottom row). (D) Reconstruction of a micrograph depicting a 3D, interconnected vascular network, evidenced by CD31 immunostaining (green). The vascular network shows the presence of lumens with an average diameter of 5.9 μm.

To form a hydrogel platform containing vascular channels, we used a protocol adapted from the literature.^20−22^ As a hydrogel, we chose the same fibrin formulation used to fabricate the PN platform to ensure permissiveness for the growth of both tissues and to facilitate integration at a later stage. In our approach, we combined HMVECs and HDFs, at a concentration of 1.5 and 0.3 M cells/mL, respectively (5:1 ratio), randomly dispersed within a fibrin hydrogel. The cells were cultured for 10 days in vessel medium (VM) to allow vessel formation and maturation (Figure 2A), for which the presence of HDFs is critical (Figure 2B) because HDFs produce angiogenic factors that stimulate vessel formation and directly associate with the vessels to stabilize them.^23−25^ Cultures lacking HDFs (Figure 2B, left) showed poorly formed and not well-interconnected vessels. In contrast, the inclusion of HDFs resulted in vessels that are better formed and display good interconnectivity (Figure 2B, right). We also characterized these newly formed vessels according to traditional phenotypical markers present in native vasculature (Figure 2C). VE-cadherin, a major component of the adherens junctions and essential for the endothelial barrier,^26^ was visibly present at the border between cells (Figure 2C, top left). Von Willebrand factor (vWF), a blood glycoprotein that mediates platelet adhesion to damage sites, essential for hemostasis and characteristic of mature vessels,^26^ was also present along the vessel wall (Figure 2C, bottom left). Laminin and collagen type IV, two extracellular matrix (ECM) molecules that are part of the endothelium basal lamina and also indicate vessel maturation,^27,28^ were present and located adjacent to the vessel wall, forming the basement membrane (Figure 2C, right column). The formed vessels were 3D and open inside (Figure 2D), with an average lumen diameter of 5.9 μm, which is slightly smaller than typically reported values,^29^ but within the range for capillaries (5–10 μm).^30^

### Formation of a 3D Vascular Platform with HMVECs and SCs

After establishing a vascular model composed of HMVECs and HDFs, we were also interested in assessing the potential of SCs as vasculogenesis promoters and mural cells. Since SCs are known to secrete VEGF and are used in our PN model to achieve myelination, the substitution of HDFs with SCs would simplify the construction of a neurovascular model. However, we first evaluated the feasibility of using rat SCs with human cells by investigating the ability of rat VEGF (VEGF-165) to bind and activate the VEGF2 receptor on HMVECs. Both human and rat VEGF at 5 ng/mL were sufficient to induce the receptor phosphorylation (Figure S1). Conversely, when using control medium (basal medium plus 5% fetal bovine serum (FBS) and depleted of VEGF), the receptor was not activated. Once we confirmed the ability of rat-derived VEGF to communicate with HMVECs, we also tested the purity of our SC population. When extracting these cells from sciatic nerves, the isolated cell population contains contaminating fibroblasts, but these can be removed to obtain pure SCs cultures, as evidenced by the ubiquitous S100^+^ cells (SC marker) and the absence of CD90^+^ cells (fibroblast marker) (Figure S2).

**Figure 3:**
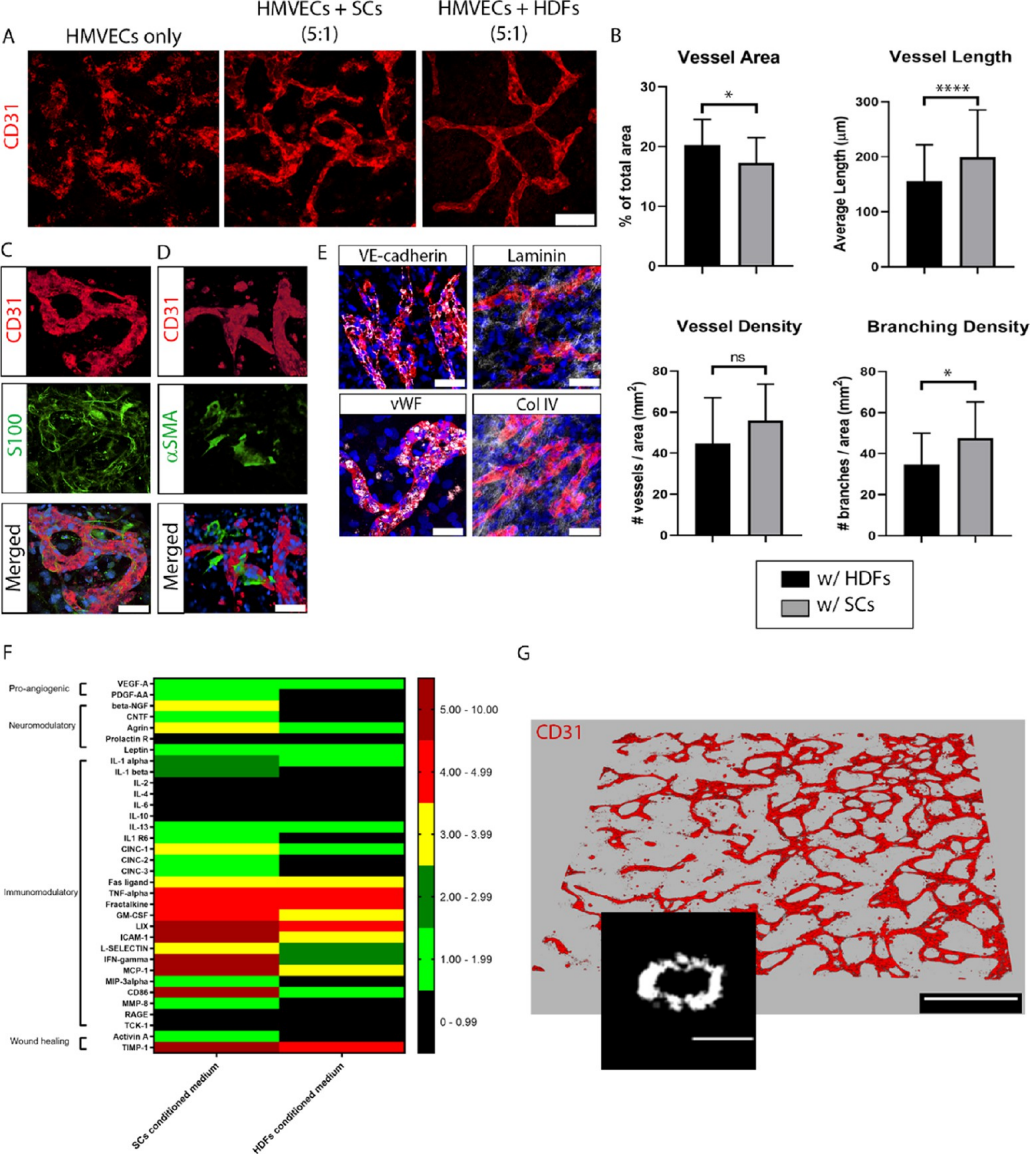
Formation and characterization of a 3D vascular channel network composed of HMVECs and SCs. (A) Comparison of vascular networks stained by CD31 (red) after 10 DIV, composed of HMVECs only (left), HMVECs plus HDFs at 5:1 ratio (middle), and HMVECs plus SCs at 5:1 ratio (right). Scale bar represents 100 μm and applies to all images. (B) Quantification of vessel area (top left), vessel length (top right), vessel density (bottom left), and branching density (bottom right) formed in co-cultures of HMVECs with either SCs (black bars) or HDFs (gray bars). The bars represent the mean ± standard deviation (SD) of two experiments using ≥4 replicates per condition. For the image analysis, we took at least five images per sample. Statistics were performed using an unpaired *t*-test, where ****p < 0.0001, *p < 0.05, and ns denotes *p* > 0.05. (C, D) Micrographs depict an intimate association between vessels (CD31; red, top row) and SCs (S100; green, middle left) that also express α smooth muscle actin (αSMA; green, middle right). The bottom row shows the merged images. Scale bars represent 50 μm and apply to panels in the same column. (E) Vascular vessels resulting from the co-culture of HMVECs and SCs stained with the traditional markers (white in the respective panels): VE-cadherin (top left), laminin (top right), vWF (bottom left), and col IV (bottom right). CD31 is shown in red and DAPI in blue. Scale bars represent 50 μm. (F) Comparison of a cytokine array present in SC- (left column) versus HDF-conditioned medium (right column). The secreted amount of each cytokine was normalized to a normal, control medium. The value of the ratio between conditioned and normal medium for each cytokine was translated to a color code, denoted 0–0.99 (black), 1.00–1.99 (light green), 2.00–2.99 (dark green), 3.00–3.99 (yellow), 4.00–4.99 (red), and 5.00–10.00 (dark red). Cytokines were organized into categories representing their most prominent function, such as pro-angiogenic, neuromodulatory, immunomodulatory, or wound healing. (G) Reconstruction of a vascular network stained by CD31 (red) showing the formation of well-defined, 3D interconnected vessels with lumens (inset, average diameter of 4.6 μm). Scale bar in the network overview represents 500 μm and in the inset, 5 μm.

Next, we co-cultured HMVECs with either HDFs or SCs in a fibrin hydrogel (Figure 2A) and evaluated the resulting vessel network after 10 DIV (Figure 3). As expected, co-cultures with HDFs promoted an extensive interconnected and stable vessel network (Figure 3A, right). Remarkably, when co-cultured with SCs, the resulting network was also well formed, interlinked, and seemingly steady (Figure 3A, middle), similar to HDFs/HMVEC co-cultures. In contrast, cultures of HMVECs alone did not produce any vessels (Figure 3A, left). Through image analysis, we quantified the vessel morphology obtained in the co-culture systems (Figure 3B). There were no significant differences in vessel density, i.e., number of vessels per area, between the two cell types (44.6 ± 22.4 in HDFs versus 55.9 ± 17.7 in SCs). HDFs promoted a greater (*p* < 0.05) vessel area than SCs (20.2 ± 4.3 versus 17.2 ± 4.3%, respectively), but a shorter vessel length (*p* < 0.001) (155.7 ± 66.1 versus 199.2 ± 85.9 μm, respectively). Finally, branching density analysis, i.e., number of branching points per area, revealed a higher number (*p* < 0.05) in SCs cultures (47.5 ± 17.6) compared to cultures containing HDFs (34.7 ± 15.3).

After the surprising finding that SCs promoted vessel development to a similar extent as HDFs, we further characterized this new co-culture system. SCs (S100^+^; green) were dispersed through the gel but also directly associated with the vessel wall (CD31^+^; red; Figure 3C). Interestingly, SCs adjacent to the vessel wall expressed α smooth muscle actin (αSMA), a cell marker indicative of mural cell differentiation^31^ and vessel maturation (Figure 3D). Differently, SCs that were not in direct contact with BVs did not express αSMA. Using the same set of markers as described above for HDFs, we further characterized the vessel phenotype (Figure 3E): VE-cadherin (top left), vWF (bottom left), laminin (top right), and col IV (bottom right) were also present and located in the same regions. In a similar fashion to HMEVCs/HDFs co-cultures, HMVECs/SCs co-cultures in fibrin (10 DIV) also resulted in a vast 3D BV network with a slightly narrower mean lumen diameter of 4.6 μm (Figure 3G). A moving cross section of the BVs (Movie S1) shows homogeneous and open vessels all throughout the network.

After validating the potential of both HDFs and SCs to induce vasculature formation, we performed a secretome analysis to compare the specific growth factors released by HDFs and SCs. The secretome profile of both cell types was similar, particularly for wound healing and immunomodulatory cytokines, with exception of IFN-γ and CD86, which were substantially higher for SCs compared to HDFs (Figure 3F). As expected, due to their role in vivo, SCs also released a larger concentration of neuromodulatory cytokines, including β-NGF, CNTF, and agrin. With regard to pro-angiogenic cytokines, VEGF-A was released in similar amounts by both cell types, but PDGF-AA was more actively secreted by SCs. The original cytokine array membrane images can be found in Figure S4.

### SC-Conditioned Medium Analysis and Influence on Vasculogenesis

**Figure 4:**
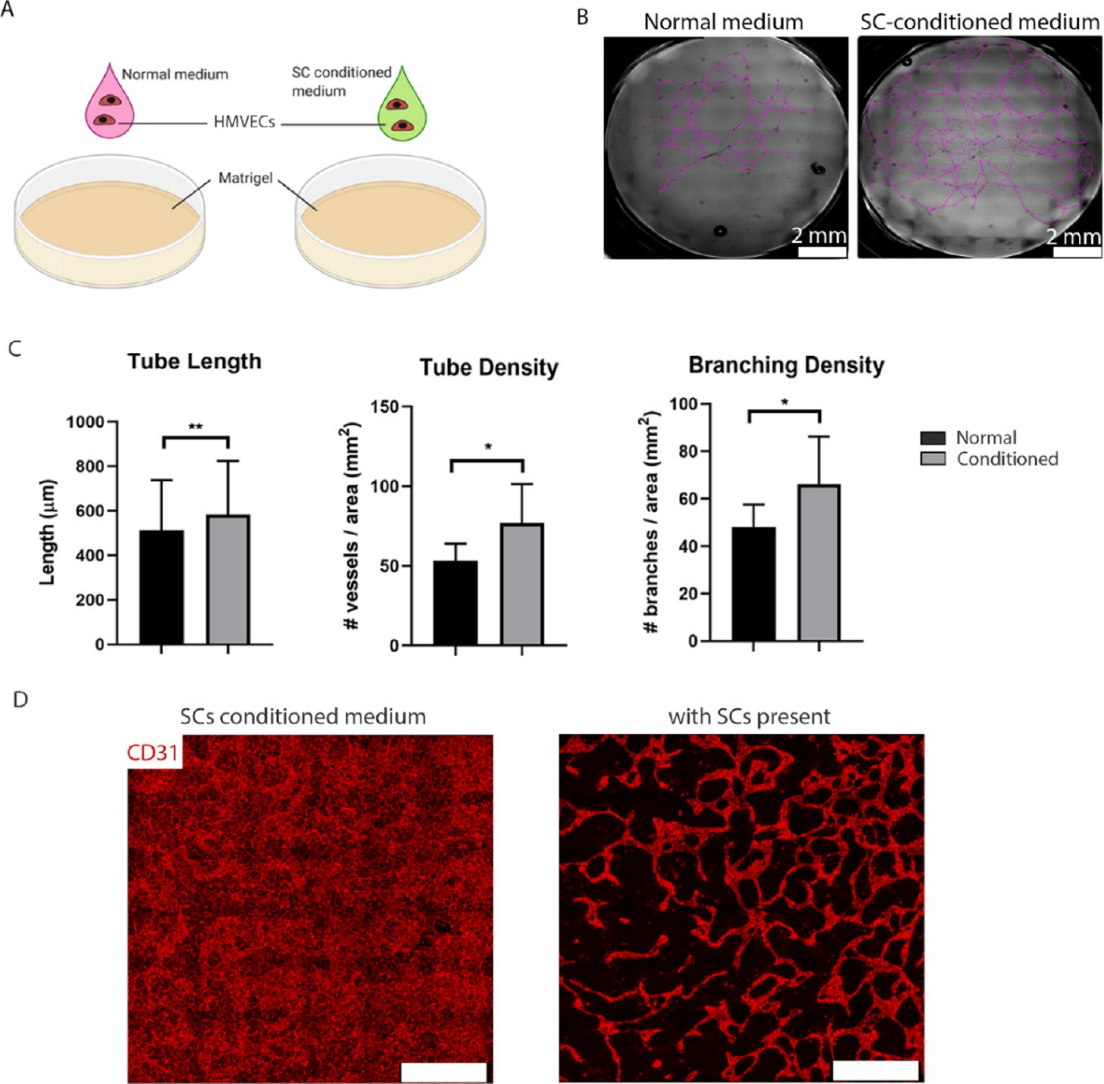
Exploring the influence of SCs on the formation of vascular networks with HMEVCs. (A) Illustration of the experiment where HMVECs were seeded in either normal or SC-conditioned medium, onto Matrigel-coated wells at 75 × 10^3^ cells/cm^2^, and the resulting network was evaluated after 48 h. Illustration made with biorender (https://biorender.com/). (B) Bright-field micrographs taken 48 h after cell seeding showing the differences in tube formation between normal (left) and SC-conditioned medium (right). Purple traces were drawn on top of the tubes for better visualization. The scale bar represents 2 mm. (C) Quantification of vessel length (left), vessel density (middle), and branching density (right) of HMEVC tubes cultured in normal (black bars) or SC-conditioned (gray bars) medium. The bars represent the mean ± SD from two independent experiments and three replicates per condition. The whole well was imaged to determine the measured parameters. Statistics were performed using an unpaired t-test, where **p < 0.01 and *p < 0.05. (D) Micrographs of 10 DIV cultures of HMEVCs in SC-conditioned medium (top) or HMVECs plus SCs in normal medium (bottom) show vessel formation (CD31, red). SC-conditioned medium was not sufficient to promote vessel formation in 3D cultures, and the presence of SCs was required for vessel formation and maintenance. Scale bars represent 500 μm.

To further investigate the role of SCs in vessel formation, we assessed if SC-conditioned medium is sufficient to induce vessel formation. First, we performed a classical angiogenesis assay on a Matrigel layer to compare vascular tube formation in HMVECs cultured in normal VM or VM preconditioned by SCs (Figure 4A). When cultured for 48 h with SC-conditioned medium, HMVEC tubule formation was greater (77.1 ± 24.2 versus 53.3 ± 10.6 tubes/mm^2^; *p* < 0.05; Figure 4C) and more widespread than with normal VM (Figure 4B). For improved visualization, we traced the tubules with a purple line (original bright-field images shown in Figure S3). Additionally, HMVECs cultured with conditioned medium produced significantly longer (583.6 ± 239.8 versus 513.3 ± 224.2 μm; *p* < 0.001) and more branched (66.3 ± 20.0 versus 48.0 ± 9.6 branching points/mm^2^; *p* < 0.05) tubules compared to HMVECs in normal VM (Figure 4C). Finally, we assessed if SC-conditioned medium was sufficient, in lieu of SCs, to produce the same angiogenic potential in a fibrin-based 3D culture with HMVECs. In cultures with conditioned medium but lacking SCs, HMVECs proliferated abundantly throughout the gel and a monolayer formed on top of the gel, but no apparent vessel formation was observed (Figure 4D, left). In contrast, the presence of SCs led to the formation of a vast interconnected network of well-delineated vessels (Figure 4D, right). Together, these observations argue that SC-preconditioned medium is sufficient for vessel formation in Matrigel but that SCs must be present for vascular network formation in a 3D culture.

### Directing Vessel Orientation through SCs Patterning

**Figure 5:**
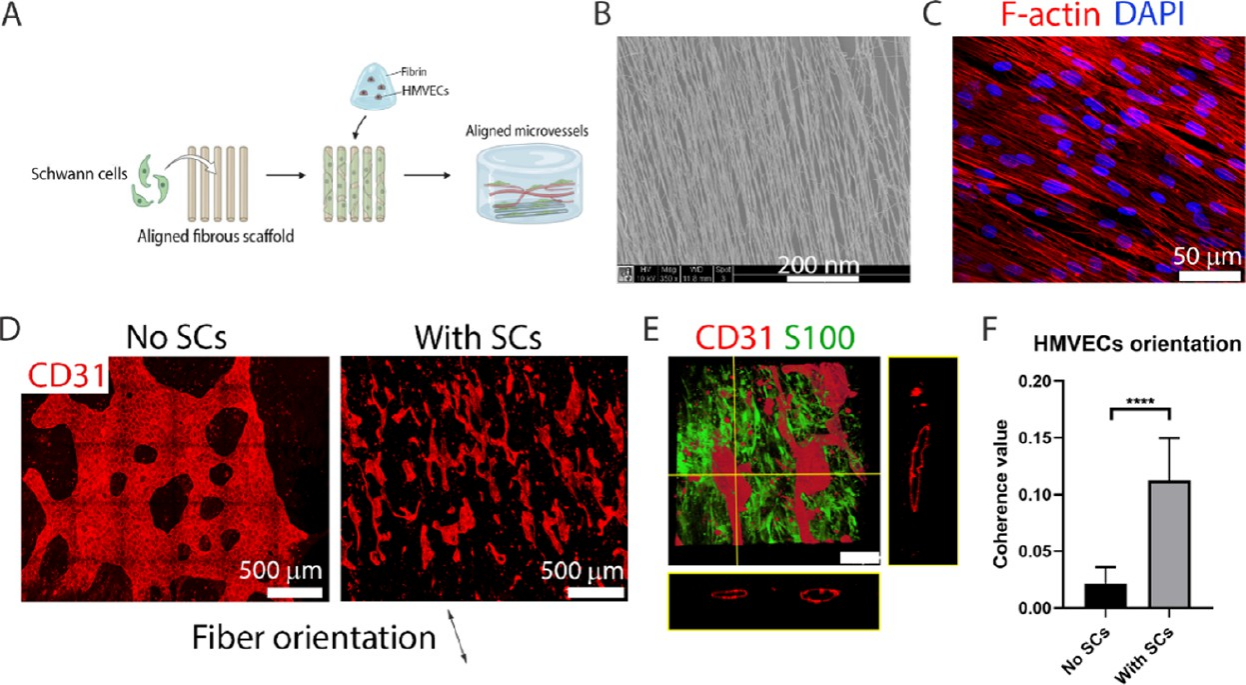
Prealigned SCs can influence HMEVCs to form an oriented vessel network following the same direction. (A) Illustration of the experiment, where 100 × 10^3^ SCs were seeded on an aligned fibrous scaffold and cultured in SC proliferation medium for 7 DIV to form aligned cell bands. The scaffold was then covered with 300 μL of fibrin containing 1.5 M HMVECs/mL. The co-culture was maintained for 10 DIV in normal VM. Illustration made with biorender (https://biorender.com/). (B) Scanning electron microscopy (SEM) image of the scaffold showing the presence of aligned microsized polymeric fibers composed of PEOT/PBT. The scale bar represents 200 nm. (C) After 7 DIV in the scaffold, SCs were highly aligned. F-actin is shown in red and DAPI in blue. The scale bar represents 50 μm. (D) Presence of prealigned SCs promoted the formation of vessels (CD31, red) that follow an overall fiber direction (right). The mean length of these vessels is 550.9 ± 265.3 μm. In the absence of SCs within the scaffold/fibrin platform, HMVECs did not form vessels or follow any overall direction (left). (E) SCs immunostained by S100 (green) associated closely at the *z*-plane of the vessels (CD31, red). The vascular channels also displayed wide lumens as seen by the CD31 orthogonal projections of the *xz*- and *yz*-planes. (F) Quantification of HMVEC orientation via coherence measurement on equally sized ROIs from CD31+ micrographs (where 0 is full isotropy and 1 is full anisotropy). The presence of SCs promoted a statistically significant increase (*p* < 0.0001) in cell alignment. The bars represent the mean ± SD from five replicates per condition. Ten measurements were taken per sample. Statistics were performed using an unpaired *t*-test, where ****p < 0.0001.

After identifying a direct role of SCs in aiding vessel formation, we questioned if these were able to induce vessel patterning. To assess this, we generated an experimental setup using SCs seeded on aligned microfibrous scaffolds to pattern SC bands. Afterward, HMVECs were seeded in a fibrin hydrogel on top of the construct, and vessel formation was evaluated after 10 DIV (Figure 5A). The scaffolds used were identical to those in the PN platform and were composed of poly(ethylene oxide terephthalate)/poly(butylene terephthalate) (PEOT/PBT) aligned microfibers of 1.37 ± 0.20 μm (Figure 5B).

SCs, seeded at 100 × 10^3^, adhered, proliferated, and aligned with the scaffold after 7 DIV, as visible by F-actin staining (Figure 5C). After the addition of a 300 μL fibrin gel containing HMVECs (1.5 M cells/mL), the formed vessels acquired an overall orientation that followed the direction of the underlying scaffold fibers (Figure 5D, right). The average length of these vessels was 550.9 ± 265.3 μm. In control cultures, with scaffolds devoid of SCs, vessel formation was minimal and most HMVECs organized in a branched monolayer (Figure 5D, left). In the latter situation, HMVECs orientation was isotropic, and thus cell alignment measurements showed a low coherence value of 0.02 ± 0.01 (Figure 5F). In contrast, SC-seeded scaffolds promoted a significantly larger (*p* < 0.0001) HMVEC anisotropy, with a coherence value of 0.11 ± 0.04. Additionally, the patterned microvessels were 3D and maintained open channels throughout their structure, as visible in the orthogonal projections of CD31^+^ cells from both viewing planes (Figure 5E). A moving cross section of the vessels (Movie S2) shows that the lumens were steadily maintained through the aligned vascular network. S100-stained SCs were present along the vessels’ border throughout the whole construct, maintaining an aligned morphology (Figure 5E) at the bottom of the construct and a random morphology at the top (Movie S3).

Compared to randomly dispersed SCs/HMVECs fibrin cultures, the vasculogenic potential of prepatterned and aligned SCs is almost identical since the EC density is the same and there are only minor differences regarding SCs seeding density (90 × 10^3^ cells in random cultures and 100 × 10^3^ cells/scaffold). However, morphological differences are vast, with the prepatterned SCs scaffold promoting longer (∼550.9 μm) and more aligned vessels, but with lower interconnectivity.

### Development of a “Combined” NV Platform

**Figure 6:**
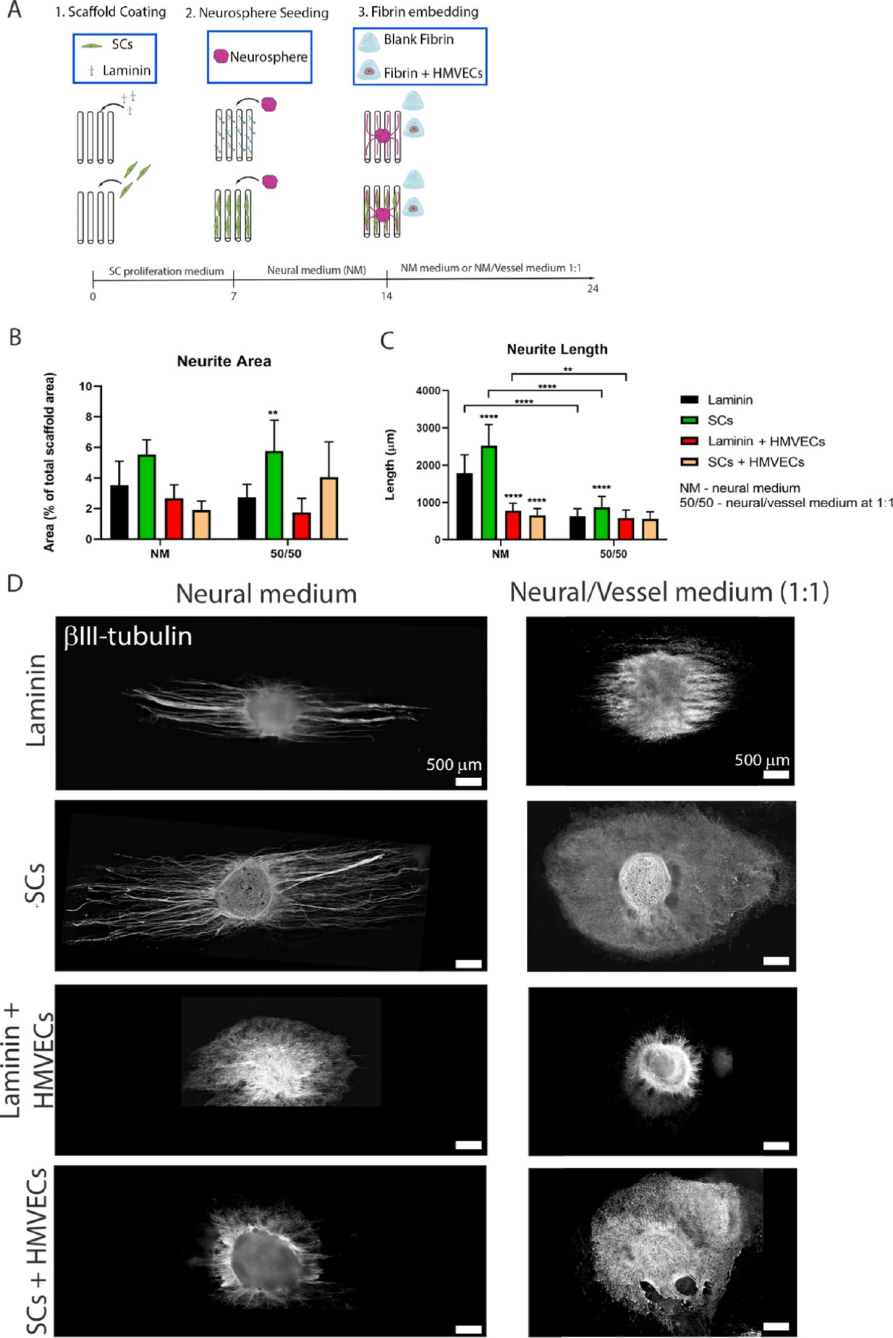
Development of the combined NV platform and neural tissue assessment. (A) Illustration of the followed strategy. Aligned microfibrous scaffolds were coated with either laminin (1 μg/mL) or seeded with SCs (100 × 10^3^ cells per scaffold). The cultures were maintained for 7 DIV in the SC proliferation medium to allow SCs to populate the scaffold. On day 7, one neurosphere was added to each scaffold. The culture medium was then switched to neural medium (NM) and maintained for 7 days to induce neurite outgrowth. On day 14, 300 μL of blank fibrin or fibrin containing 1.5 M HMVECs/mL was added and the cultures were maintained for 10 DIV in either full NM or a 1:1 mixed NM and VM. Illustration made with biorender (https://biorender.com/). Quantification of the neurite area (B) and neurite length (C) between samples of laminin-coated scaffolds plus blank fibrin (black bars); SC-seeded scaffolds plus blank fibrin (green bars); laminin-coated scaffolds plus HMVEC-laden fibrin (red); or SC-seeded scaffolds plus HMVEC-laden fibrin (light orange bars). The samples were cultured in NM (left set of bars) or 1:1 NM/VM (right set of bars). The bars represent the mean ± SD from at least five replicates per condition in two independent experiments. The whole sample was measured to quantify the neurite area and neurite length (see the Methods section). At least 20 measurements were taken per sample. Statistics were performed via two-way analysis of variance (ANOVA) followed by Tukey’s multiple comparison test, where ****p < 0.0001 and ** *p* < 0.01. Comparisons were done relative to laminin coating and blank fibrin samples (for each medium group). (D) Neurite morphology after culture in the eight different platform conditions, via immunostaining to βIII-tubulin (white). Scale bars represent 500 μm.

Following the development of a 3D neural platform and a 3D microvascular model, we set out to combine both biofabrication approaches to create a 3D NV platform (Figure 6A). We compared the neural growth and vascular development in platforms with different compositions, such as the scaffold coating (1 μg/mL laminin or 100 × 10^3^ SCs) and fibrin content (blank or with HMVECs), and cultured with different media, either pure neural medium (NM) or neural/VM in equal proportions (1:1 medium). In this way, we could dissect the influence of individual parameters and determine the best conditions for optimal neural growth and vascular development. Initially, scaffolds were cultured for 7 DIV in an SC proliferation medium, allowing SC proliferation and alignment. After that, one neurosphere was added and the platforms were cultured for a further 7 DIV in a neural medium to promote neurite outgrowth. On day 14, a blank or HMVEC-laden (1.5 M cells/mL) fibrin hydrogel was embedded in the construct. The final combined platforms were further cultured for 10 DIV in NM or 1:1 medium. In all conditions, the neurons attached and survived the culture period and their growth parameters are described in Figure 6.

#### Neural Tissue Formation

Quantification of neurite area as a percentage of neurites occupying the total scaffold area (Figure 6B) showed that the greatest growth occurred in SC-seeded scaffolds with blank fibrin (green bars), which occupied 5.5 ± 0.9% in NM (area is 6.24 μm^2^) and 5.8 ± 2.0% in the 1:1 medium (area is 6.70 μm^2^). Laminin-coated scaffolds with blank fibrin (black bars) displayed 3.5 ± 1.6% for NM (area is 3.97 μm^2^) and 2.7 ± 0.9% for 1:1 medium (area is 3.08 μm^2^). For samples containing HMVECs, laminin-coated scaffolds (red bars) led to an area of 2.6 ± 0.9% for NM (area is 3.00 μm^2^) and 1.9 ± 0.9% for 1:1 medium (area is 1.96 μm^2^), while SC-seeded scaffolds with HMVEC-laden fibrin (light orange bars) resulted in 1.9 ± 0.6% occupancy in NM medium (area is 2.14 μm^2^) and 4.1 ± 2.3% when in the 1:1 medium (area is 4.58 μm^2^).

For each medium group, there was a similar trend regarding neurite length in the different scaffold conditions (Figure 6C). Neurons cultured with NM developed longer neurites than in the 1:1 medium regardless of the coating/seeding. SC-seeded scaffolds with blank fibrin (green bars) promoted the highest neurite length, with a mean value of 2522.4 ± 564.2 μm in NM and 868.3 ± 294.5 μm in the 1:1 medium. Laminin-coated scaffolds with blank fibrin (black bars) promoted a mean length of 1784.0 ± 491.4 μm when cultured with NM, and 624.2 ± 207.8 μm when cultured in the 1:1 medium. For fibrin gels containing HMVECs, neurite outgrowth was reduced significantly (*p* < 0.0001) in the NM group for both laminin-coated (775.3 ± 203.7 μm) (red bars) and SC-seeded scaffolds (651.9 ± 184.1 μm) (light orange bars), compared to laminin scaffolds with blank fibrin (black bars). When using the 1:1 medium, the difference from HMVEC-containing samples to blank fibrin conditions is practically negligent, with laminin-coated scaffolds (red bars) resulting in 577.7 ± 139.7 μm and SC-seeded scaffolds (light orange bars) resulting in 557.0 ± 62.2 μm. A visual depiction of the neurite morphology of these samples is presented in Figure 6D.

Together these data allow us to extrapolate the effect of the different parameters—medium, scaffold coating, and fibrin content—on the forming neurite network. NM medium seems to favor neurite outgrowth compared to 1:1 medium, while neurite area was similar in scaffolds with the same content but cultured in different media. Regarding the fibrin content, HMVEC-laden gels surprisingly induced a generalized decrease in area and length compared to blank fibrin-composed samples. Finally, the influence of SC-seeding versus laminin coating was highly notable, with SCs promoting higher neurite area and length.

#### Vascular Tissue Development

**Figure 7:**
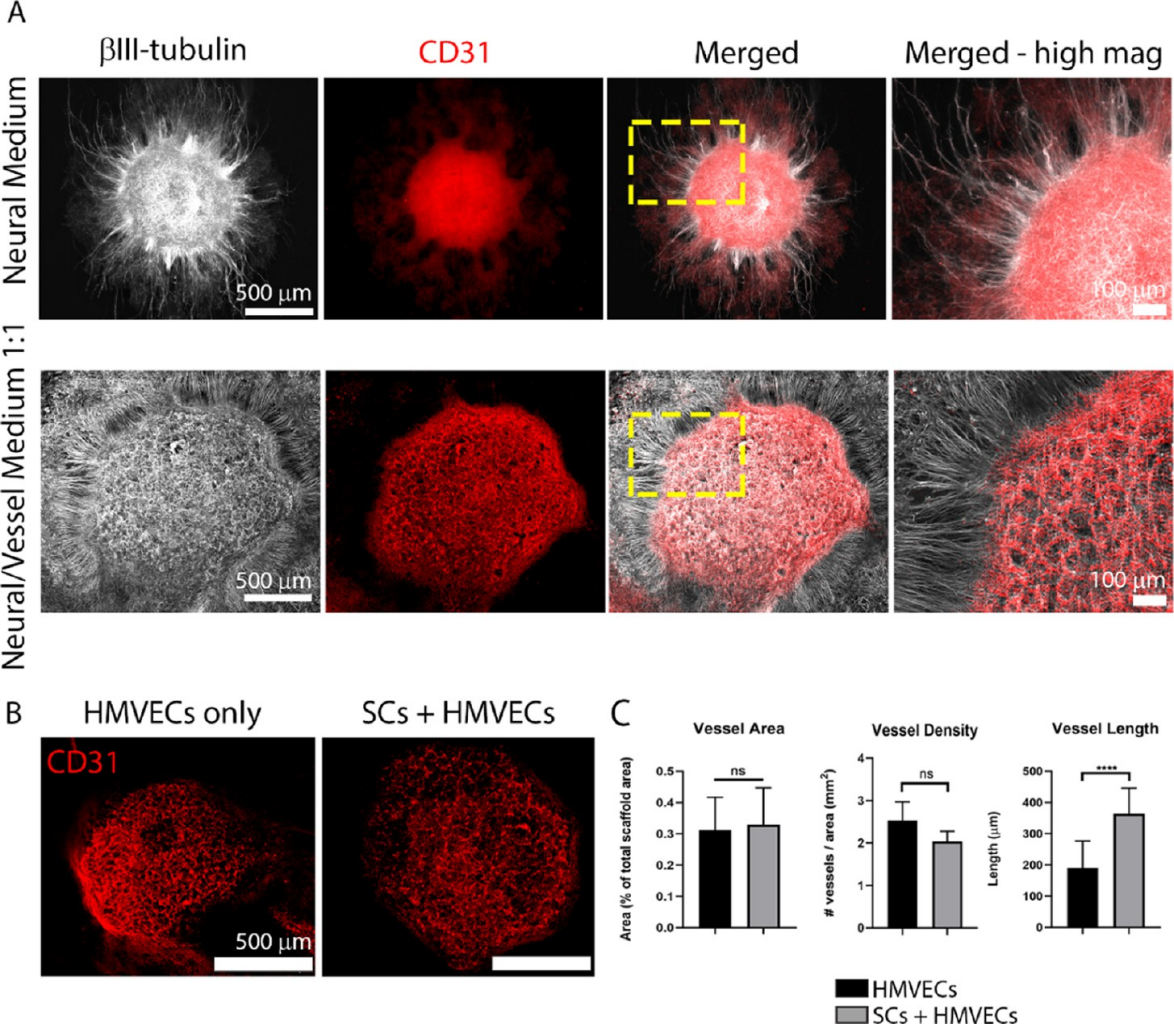
Vascular tissue development in the combined NV platform under different conditions. (A) Comparison of the neural and vascular tissue formation on platforms composed of SC-seeded scaffolds and hMVEC-laden fibrin cultured on neural medium (NM, top row) or 1:1 NM/VM (bottom row). NM promoted neurite outgrowth but was not sufficient to induce vessel formation, whereas 1:1 medium promoted simultaneous neuron proliferation, neurite outgrowth, and vessel formation, which was mostly circumscribed to the top of the neurosphere. Neurospheres labeled with βIII-tubulin (white) and vessels by CD31 (red). The images in the far right column show a high-magnification view of the yellow-dashed box marked in the merged column. Scale bars in the far left images represent 500 μm and apply to the two images to their respective right; scale bars in the far right images represent 100 μm. (B) Vessel formation (CD31, red) was similar on laminin-coated scaffolds (left) or SC-seeded scaffolds (right). Scale bars represent 500 μm. (C) Quantification of the vascular network on platforms with (gray bars) and without (black bars) SCs using the parameters: vessel area (left graph), vessel density (middle graph), and vessel length (right graph). The bar graphs represent mean ± SD from at least five replicates per condition in two independent experiments. Statistics were performed using an unpaired *t*-test, where ****p < 0.0001 and ns denotes not significant.

For the conditions containing HMVECs, an analysis of the vascular development is provided in Figure 7. In all conditions, we found HMVECs (immunostained by CD31) were in close vicinity to the neuron cluster (βIII-tubulin^+^) and seemingly attracted to it, regardless of medium composition and scaffold content (Figure 7A). Medium composition was however determinant to the resulting vascular morphology. When using NM (Figure 7A, top), HMVECs were unable to form vessels, organizing instead into a monolayer that covered the whole neuron cluster and even expanded beyond it. On the other hand, in the 1:1 medium (Figure 7A, bottom), HMVECs were able to form an interconnected BV network, circumscribed to the neuron cluster, and showing an overall architecture clearly congruent with the neurite network.

To investigate the effect of scaffold coating, i.e., SC addition, to vascular development in NV platforms, we analyzed the vessel density, area, and length between laminin-coated and SC-seeded samples, cultured in the 1:1 medium (Figure 7B,C). Since NM did not yield any vascular structures, we excluded these samples from this quantification. The vessel area was identical between the two conditions, displaying an occupancy of 0.3 ± 0.1%. Vessel density was superior, although not significantly, in conditions without SCs (2.5 ± 0.4 vessels/mm^2^) compared to those with SCs (2.0 ± 0.2 vessels/mm^2^). Finally, regarding vessel length, we detected a significant difference (*p* < 0.0001) between conditions, with platforms containing SCs exhibiting longer BVs with 363.8 ± 82.2 μm compared to those absent of glial cells (190.4 ± 86.5 μm).

Taken together, these findings suggest that medium composition is crucial for vasculature formation, with NM, a medium optimized for neural growth, not permitting BV formation, whereas the 1:1 medium is already sufficient to promote the emergence of vasculature. Contrary to what we have seen before, SC addition in the NV platform does not seem to produce a significant impact on BV formation. In platforms absent of SCs, BVs were still able to form and had a similar area and density, but shorter length, than in SC-containing platforms.

### Optimized “Segregated” NV Platform

**Figure 8:**
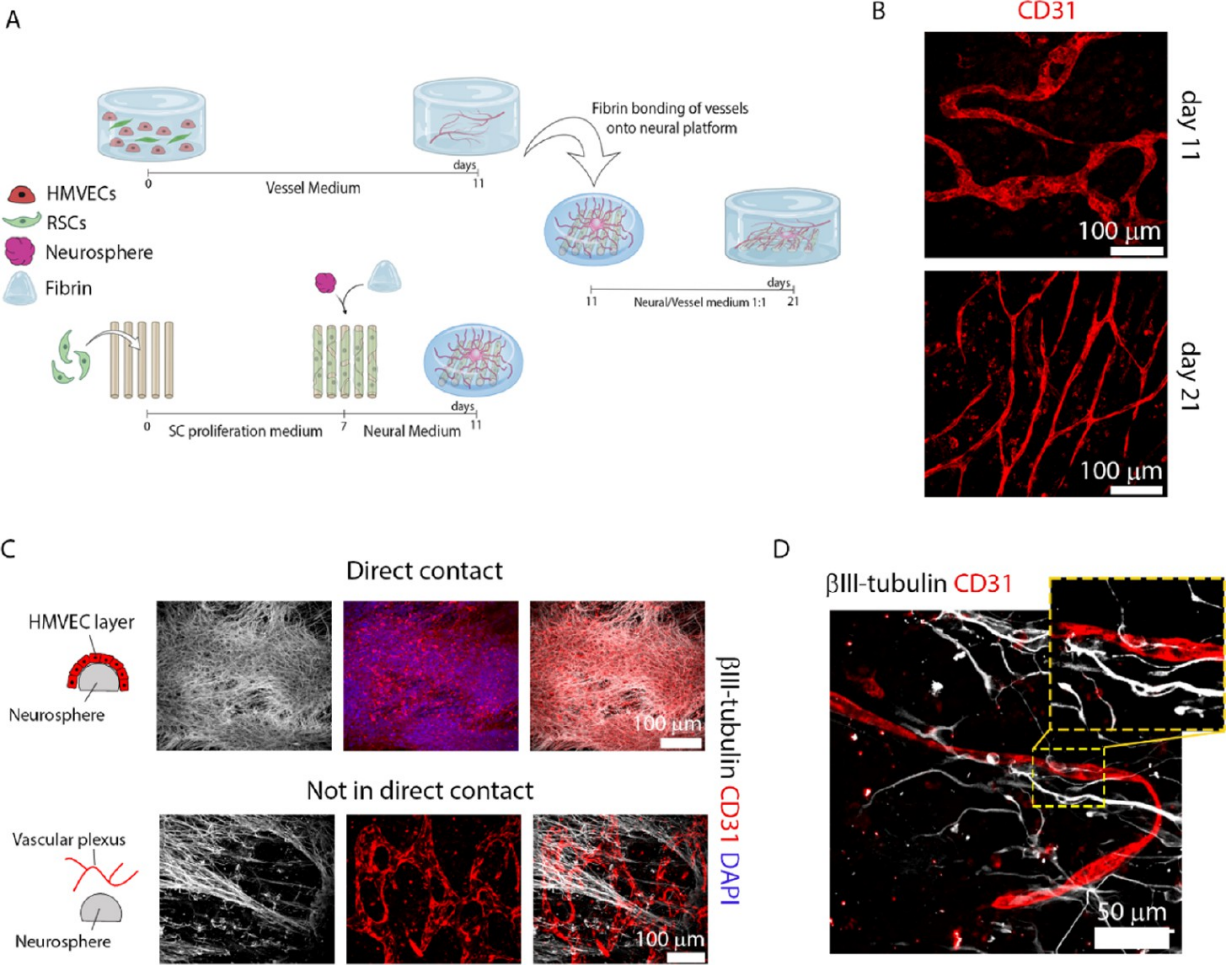
Development and characterization of the segregated NV platform. (A) Illustration of the platform and assembly method via integration of two separate components: a 3D vessel network platform (top) and a 3D neural tissue platform (bottom), both at 11 DIV. The segregated NV platform is cultured for further 10 DIV in the 1:1 medium (see the Methods section for further details). Illustration made with biorender (https://biorender.com/). (B) Vessel morphology (shown by CD31 in red) in the NV platform at 21 DIV (bottom) shows further maturation denoted by thinning, less branching, and a straighter pattern compared to vessels at 11 DIV from the vascular-only model (top). Scale bars represent 100 μm. (C) Vascular development in the vicinity of the neurosphere, within the segregated NV platform (21 DIV). HMVECs (immunostained by CD31, red) were attracted to the neurosphere (immunostained by βIII-tubulin, white) and formed either a monolayer when in direct contact (top row) or a vascular plexus when not in direct contact (bottom row). Right, merged images. Nuclei stained with DAPI (blue). Scale bars represent 100 μm and apply to the panels in the respective row. (D) Vessel innervation occurred primarily at regions farther away from the neurosphere cluster, where neurites (βIII-tubulin, white) could innervate and align with the vessels (CD31, red) as highlighted in the yellow-dashed box, zoomed in the top right corner box. Scale bar is 50 μm.

In the combined NV platform (Figures 6 and 7), the NV tissue formation is suboptimal compared to the individual cultures described for neurons (Figure 1) and for vessels (Figures 2 and 3). Regarding the neural component, neuron outgrowth was hindered by HMVEC presence (Figure 6), while the vasculature shown in Figure 7 was visibly less morphologically mature than vascular-only model (Figure 3). For this reason, we sought an improved strategy, where both tissues are first segregated and cultured individually during an initial period, in their optimized conditions, and bonded at a later developmental stage to form into a single unit composed of the two segregated NV components (Figure 8A).

The nerve component was prepared as described in Figure 1, with the iPSCs/SCs co-culture maintained for 4 days, at which point we observed vast neurite outgrowth from the cluster. The vascular component contained a co-culture of HMVEC/SCs, built as described in Figure 3, and cultured for 11 DIV which allows cell organization and growth into an interconnected vessel network. To bond both structures, we added fibrin gel, which is a common material between both models, and thus promoted facile integration. After 10 DIV of NV culture in the 1:1 medium (meaning 21 DIV for both subunits), we investigated the developed neuronal morphology/vasculature and resulting NV interactions. To compare the progression of vascular development in platforms pre- and post-integration with the neural scaffolds, we assessed vascular morphology in 11 DIV (pre-integration; vascular component) and 21 DIV cultures (post-integration; NV platform). The 11 DIV co-cultures showed well-formed vessels, reminiscent of a vascular plexus, with a large diameter, short length, and a tortuous/meandering pattern. In 21 DIV co-cultures, the vessels continued to develop and the resulting structures resembled mature microvessels, with a thinner diameter, larger length, and more strongly oriented (Figure 8B). Quantification of the vessel networks from 11 to 21 DIV showed the mean vessel diameter decreased from 31.1 ± 9.5 to 9.2 ± 2.3 μm (Figure S5A; *p* < 0.0001), while the mean vessel length increased from 199.2 ± 85.8 to 279.5 ± 140.6 μm (Figure S5B; *p* < 0.001). Neither the vessel area (Figure S5C) nor branching density (Figure S5E) differed between timepoints. However, a greater number of vessels per area (Figure S5D) was observed at 21 DIV (111.9 ± 27.4 vessels/mm^2^) compared to 11 DIV (56.0 ± 17.8 vessels/mm^2^).

In the segregated NV platform (21 DIV), we found a large presence of HMVECs in the region of the neurosphere (Figure 8C), which signifies their attraction to the neuron cluster. HMVECs that migrated and came in direct contact with the neurosphere (Figure 8C, top images) tended to cover its whole surface, forming a monolayer, with no signs of BV formation. In a different manner, HMVECs that were attracted to this region, but remained over 200 μm from the neurosphere surface were able to form vascular structures. These structures, resembling a vascular plexus, appeared to be newly formed, due to their morphology that was visibly less mature than other microvessels found within the 21 DIV NV platform, as evidenced in Figure 8B. In regions distant from the neurosphere, we observed a large presence of mature vessels and no indication of new vessel formation or EC migration (Figure 8D). Neurites that extended over large distances into these remote regions were observed to surround the microvessels located there. In several cases, we noticed congruency of NV tissue, as denoted by the parallel and often overlapping architecture of axons and BV network (Figure 8D).

## Discussion

Most organs are dependent on innervation and vascular irrigation for their normal function and homeostasis maintenance. For this reason, the move toward increasingly biomimetic, functional, and complex in vitro organ models will be reliant on the appropriate integration of NV tissue within these models. Interactions between neurons, glial cells, and endothelial cells are also crucial for the formation of the stereotyped NV axis, starting during the developmental phase and persisting in adult life. Additionally, several pathologies, such as diabetes type II, produce NV dysfunctions, which can cause severe disruptions in overall organ function.^20,32,33^ To further our understanding regarding the NV ecosystem, in vitro NV models can provide a precious help, by offering a research tool that allows us to investigate and interrogate NV interactions, decode the specific biological players, and determine pathophysiology mechanisms. To date, some efforts have been carried out to reconstruct the NV multicellular ecosystem, although with limitations regarding the relevance of cell sources and level of biomimicry.^16−18,34^

Here, we have developed an NV platform that takes into consideration the crucial biological and physical requirements of the neural and vascular components, by integrating an optimized 3D nerve model and an optimized 3D vascular network model, into a single cohesive unit. To form a nerve model, we used our previously reported method,^19^ in which iPSCs-derived nociceptor neurospheres are co-cultured with rat SCs in a polymeric fibrous scaffold and embedded in a fibrin hydrogel, to yield a large-scale 3D anisotropic and myelinated neural tissue (Figure 1). For scaffold fiber fabrication we used PEOT/PBT, a biocompatible, biodegradable, and cell-adherent material that does not require further chemical modifications and has demonstrated success in nerve tissue engineering applications.^35,36^ PEOT/PBT is also cheap, commercially available, and can be easily processed into aligned fibers via electrospinning (ESP) on a rotating mandrel. To emulate the nerve microenvironment and permit 3D neurite outgrowth, we embedded the cell-seeded scaffold with fibrin, a natural material involved in nerve regeneration. Fibrin is cheap, does not necessitate functionalization, and has been widely used in the fabrication of PN models.^37−39^ Furthermore, fibrin has also been successfully employed in the formation of in vitro vascular models,^40−42^ thus being ideal for the fabrication of an NV platform. To replicate the nerve cellular milieu and achieve myelination, we co-cultured the neurons with rat SCs. SCs can be obtained in high yield from rat sciatic nerves and easily purified, as well as show the ability to stimulate axonal growth and myelinate human neurons.^36,43^ For practical and economic reasons, we opted to use rat SCs in this platform, forming a mixed-species model. Despite the biological improvement provided to the neural tissue, we recognize that a switch to a human-derived population is needed to further improve the model biological relevance. Neural models using human primary^44^ or iPSCs-derived SCs^45^ have already been reported, thus future modifications to this platform will aim to establish an all-human cell system.

To produce a 3D vascular model we based our method on previous reports that combined ECs at high density and a support cell type, within a hydrogel^25^ (Figure 2A). The high EC density aids in vessel formation by replicating the initial stages of vasculogenesis, where blood islands form and fuse to give rise to the primary vascular plexus.^46^

As an EC population, we opted for primary HMVECs, which have been previously shown to generate morphologically mature vessels.^25^ These cells are obtained from skin microvessels, which form interactions with nerves in their native environment,^47,48^ thus making this model more representative and clinically relevant than HUVECs^16^ that originate from a noninnervated source (the umbilical cord). In addition, HMVECs can be directly harvested from patients, via simple skin biopsies, which also allows the fabrication of patient-specific models for precision medicine.^49^ Alternatively, stem cell-derived ECs are a possible option, but the current protocols necessitate further optimization to yield phenotypically mature vessels at the same level as primary ECs.^50^

To provide trophic support and physically stabilize the vessels at a later stage of development, support cell types such as mesenchymal stem cells,^31^ pericytes,^51^ and fibroblasts^25^ have been widely used. We opted for fibroblasts (dermal origin, HDFs) because these cells are present within native skin and nerves, thus representing a suitable partner for HMVECs and neurons. Fibroblasts secrete a myriad of soluble angiogenic factors such as VEGF and fibroblast growth factor (FGF),^52^ and several reports have shown that they are suitable partners for ECs to generate mature vasculature.^21,23,25,53^ When co-cultured with HMVECs in a fibrin hydrogel, we were able to obtain a 3D interconnected BV network with open channels, exhibiting the presence of important characteristic BV markers (Figure 2), which denotes proper vessel formation.^30,54^

SCs have also shown the ability to interact with ECs, improving their migration^10^ and directing the BV patterning in the skin.^9^ Currently, there is a scarcity of in vitro models containing SCs and ECs, and thus the specific interactions between these cell types are generally unexplored. Replicating the method employed with HDFs, we were also able to generate 3D interconnected, lumenized, and phenotypically mature BV networks using co-cultures of SCs and HMVECs (Figure 3). To the best of our knowledge, this is the first time that the vasculogenic potential of SCs is shown in in vitro cultures with ECs. Additionally, we demonstrate that SCs act as proper mural cells by directly associating with the vessels to provide a physical support (Figure 3C). More interestingly, these SCs in intimate contact with the vessel wall, and only these express αSMA (Figure 3D), a mural cell marker (expressed by pericytes and smooth muscle cells) responsible to regulate contractility in capillaries.^55^ This finding highlights that SCs, well known for their plasticity,^56,57^ may also act as pericytes, physically and chemically supporting vascular channels. Furthermore, this finding suggests the versatile nature of SCs in the context of NV platform generation, which renders the use of fibroblasts unnecessary to simultaneously obtain mature neurons and BVs.

To better understand the mechanism underlying SC mural activity, secretome analysis of SC-conditioned medium was compared to HDF-conditioned and control medium, revealing that both conditioned media contained VEGF-A, a major pro-angiogenic factor, and tissue inhibitors of metalloproteinases (TIMP)-1, a potent angiogenic inhibitor (Figure 3F).^58,59^ This confirms that a balanced production of pro- and antiangiogenic proteins is necessary for the orchestration of vascular networks and that the presence of secreted inhibitors in this case does not negate the angiogenic/vasculogenic potential of either HDFs or SCs. Additionally, we observed that SC-conditioned medium possessed a richer composition than the one obtained with HDFs, particularly higher quantities of neuromodulatory and immunomodulatory cytokines, which highlights the versatility of SCs in performing supportive roles to both neurons and ECs. Further exploring the action of SCs on HMVECs, we detected a positive influence of SC-conditioned medium on the extent of tubule formation, thus validating the role of SC-secreted cytokines in the stimulation of angiogenesis/vasculogenesis (Figure 4A–C). Conversely, Huang et al.^60^ described that human SC-conditioned medium inhibits angiogenesis, which is probably mediated by TIMP-2, a member of the tissue inhibitors of metalloproteinases (TIMP) family with known in vivo and in vitro antiangiogenic properties.^61,62^ We believe that discrepancies between our results and this can be attributed to differences in experimental conditions, which may have altered the secretome, such as the cell source and culture parameters (e.g., FBS concentration).

Finally, we tested if SC-conditioned medium was sufficient to drive vessel formation in 3D cultures in fibrin, or if their presence was required. As illustrated in Figure 3E, the presence of SCs is essential to attain a proper vessel morphology, probably due to increased and local secretion of cytokines, as well as physical interaction with HMVECs, to shape and stabilize vessels in this 3D environment. After establishing the angiogenic potential of SCs, we questioned if these cells also had the potential to drive BV orientation. In vivo reports show that BVs align with nerves, mimicking their pattern, in a VEGF-mediated process and that SCs are, together with neurons, the producers of this VEGF source.^9,63^ As previously demonstrated, rat VEGF is able to stimulate HMVECs (Figure S1) and SCs are able to induce randomly oriented vessels within fibrin hydrogels (Figure 3). Taken this together, it is unsurprising that prepatterned and aligned SCs are able to induce vasculature orientation in 3D (Figure 5), replicating the in vivo patterning role of SCs on ECs.

After establishing and characterizing the individual nerve and vascular models, we were in conditions to assemble these components into a single NV unit and study the effects of the constituents on NV tissue development (Figures 6 and 7). The culture medium is a key component to the development of any in vitro tissue, as it contains the essential nutrients that are required to maintain cell viability and stimulate cell growth and development, in similar conditions to the in vivo milieu. As expected, NM—the optimized medium for nerve growth—led to a higher neurite length for all scaffold conditions compared to a 1:1 blend of NM and VM (Figure 6C,D), but in terms of neurite area, both media produced similar results.

However, visual inspection of the neurite area suggests that the 1:1 medium may have also promoted neuron proliferation (Figure 6D); these samples contained a larger neuron population but with smaller fibers compared to NM, thus approximating the overall neurite area. This effect can be explained by the growth factors present within the 1:1 medium, such as epidermal growth factor,^64^ fibroblast growth factor,^65^ hydrocortisone,^66^ insulin-growth factor,^67,68^ and VEGF,^69^ which have been shown to induce neural stem cell proliferation. In contrast, the incorporated SCs secrete several neurotrophic factors such as NGF (as seen in Figure 3F), which explains the beneficial effect in terms of neurite length and area brought by SC-seeding on the scaffold (Figure 6B,C). But besides this, SCs also produce endogenous VEGF (as also demonstrated earlier) and, in combination with the exogenous source present in the 1:1 medium, this may further induce neuron proliferation and/or migration.^70^

Surprisingly, the addition of an EC population in our platform did not significantly enhance neurite length and area. This could be a result of EC attraction to the neurosphere, which may have hindered neurite outgrowth in favor of EC–neuron interactions. Conversely, in the 2D co-cultures of DRGs and HUVECs reported by Grasman et al.^16^ and in 3D co-cultures of ESCs–motor neurons and iPSCs–ECs described by Osaki et al.,^18^ the presence of ECs enhanced neurite outgrowth. We hypothesize that differences in the timing of EC addition, neurosphere size, and dimensionality of the construct (2D vs 3D and platform dimensions) may all contribute to these disparities.

Regarding BV formation, we detected that NM alone was not sufficient to drive vasculogenesis, whereas the mixed medium led to the formation of a linked and extensive vessel network, again with a strong spatial correlation to the neuron cluster (Figure 7A). This finding highlights the need to provide a specific and balanced chemical milieu to be able to simultaneously generate neural and vascular networks. Concerning HMVEC agglomeration within the neurosphere, we hypothesize that it is the result of a gradient of angiogenic GFs, produced by the neurons as a consequence of hypoxia. In the region of the cluster and especially in its core, the oxygen concentration is presumably lower due to cell crowding. It is well described that hypoxic conditions activate hypoxia-inducible factor-1 (HIF-1) and lead to VEGF upregulation.^71,72^ Thus, we believe that a higher VEGF concentration in the cluster region caused HMVEC attraction. This hypothesis also explains why HMVEC-only platforms could form vessels with similar area and density (but not length) as those containing HMVECs and SCs (Figure 7B,C).

We assume that neuron-derived VEGF production was probably enough to promote vessel formation, rendering SCs redundant for this process. This finding is somewhat contradictory to what we discovered previously regarding the vasculogenic benefits of SC addition and could signify that the formation of an NV platform is only reliant on two major cell types—neurons and ECs—provided that there is sufficient VEGF to drive BV formation. However, this only applies to the early stages of vasculogenesis, since it is well established that the accomplishment of mature vasculature is dependent on the recruitment and association of mural cells.^54^ In contrast with our earlier demonstration that SCs fulfill this mural cell role (Figure 3), we noted a lack of physical association between incorporated SCs and HMVECs in our initial combined NV platforms. We hypothesized this SC mural association was probably hindered by early EC–neuron interactions that physically blocked the stabilization/promoter effect brought by the glial cells. This is supported by the observed vessel morphology in this platform, which was clearly more immature than those formed in the 3D vascular model (Figure 3).

With this in mind, we developed a different biofabrication process (Figure 8A), where the neural and vascular components were formed separately under optimal conditions to allow proper tissue development and maturation. Then, at a later stage, both segregated components were merged into a single NV unit, mimicking more accurately the developmental process in which the capillary plexus is first formed from coalescent BVs and then peripheral axon innervation occurs to drive consequent NV alignment.^73^ In this segregated NV platform, the resulting BVs were thinner, longer, and forming a less branched pattern compared to BVs from the 11 DIV vascular components (Figure 8B). This observation signifies that after NV platform integration and the switch from VM to 1:1 medium, the vasculature continued to remodel and acquired a configuration typical of a stable/mature network^54^ that is clearly distinct from the primitive plexus of 11 DIV cultures. Compared to the vasculature in the combined NV platform (Figure 7), this new assembly method resulted in a more biomimetic and mature vascular tissue, even despite the shorter culture period. While longer culture periods would further improve the morphology and functionality of this in vitro model,^54^ these results establish that the initial stages of vasculogenesis are largely dependent on optimal culture conditions for primitive plexus formation and that such a nascent vascular network can further develop in combination with neurons and in a medium that suits both tissues.

A critical finding in our development of an NV platform was our observation that HMVECs were attracted to the neurosphere, formed a monolayer when in direct contact with it, and failed to form new vessels. We believe that this is resultant from the lack of available growth space and early association/interaction with neurons. When not in direct contact with the neurosphere, HMVECs had the conditions to agglomerate and form a vascular plexus of primitive morphology. This finding suggests that the preestablished vascular network is dynamic and can engage in angiogenic processes,^54^ expanding and colonizing the platform, particularly in the regions with higher oxygen/nutrient demand. In the periphery of the cluster, most BVs had a mature morphology, probably resulting from remodeling of the preestablished vascular network. In their vicinity, we could find long neurite projections farther away from the cluster, which followed the same direction as the BVs to navigate side by side (Figure 8D).

Besides NV alignment, we also found instances of juxtaposed neurons and BVs, indicating possible innervation. This finding suggests that the BV network is also exerting its influence on the neural tissue, recruiting neurites and forming a mutual arborized pattern in the process. The tissue landscape that we could naturally achieve here is reminiscent of native NV units found within organs, such as the ear skin^48^ or gut mesentery.^74^ Further improvements could lie on increasing the culture period to allow extra tissue development, further optimization of culture medium to accommodate mutual NV tissue needs, and ultimately a connection to a perfusion pump to provide flow within the vessels that induces a superior BV maturation.^54,75^ Despite this, the platform here described aims to provide the same level of biological complexity as in vivo models but in an in vitro setting, where recurrent animal sacrifice is avoided and observations can be made in a simple, direct, and affordable manner. Because the cellular environment is defined precisely, NV interactions concerning developmental and adult stages can be dissected, and the specific biological players investigated. Moreover, the control over the chemical milieu permits the simulation of pathological conditions (e.g., hyperglycemia, hypoxia, dyslipidemia) that produce NV disruptions, as well as the experimentation of therapeutic drug candidates. Finally, this platform can serve as delivery method of neural and vascular supply to a target tissue to engineer complex organ models.

## Conclusions

In sum, we propose here a method to fabricate mature, dynamic, and mutually interactive NV tissue. Particularly for the vascular component, we also showed, for the first time, the ability of SCs to function as mural cells, forming, directing, and supporting mature vasculature. We believe that the platform here described offers an advanced, versatile, and useful tool for the development and research of NV tissue in healthy and pathological conditions.

## Methods

### Agarose Microwell Mold Fabrication

A 3% (w/v) sterile agarose (Thermo Fisher Scientific) solution was prepared in phosphate-buffered saline (PBS), 8 mL of which were poured onto an in-house fabricated poly(dimethylsiloxane) (PDMS) stamp with the negative template of 1580 microwells with 400 μm diameter. Centrifugation at 845*g* was performed to remove air bubbles, followed by chilling for 45 min at 4 °C for agarose solidification. When solid, the agarose blocks were removed, cut to fit in a 12-well plate, washed with 70% ethanol, then washed twice in PBS and left at 4 °C until further use. The day before cell seeding, PBS was replaced with culture media containing advanced Roswell Park Memorial Institute (RPMI) 1640 supplemented with 1× glutamax (Thermo Fisher Scientific) and kept in the incubator at 37 °C, 5% CO_2_ overnight.

### Cell Culture

Human iPSC line LUMC0031iCTRL08 (provided by the iPSC core facility of Leiden University Medical Center) was cultured on Geltrex-coated dishes at a density of 10 × 10^3^ /cm^2^ in mTESR1 medium (Stem Cell Technology). The cells were fed every alternate day with completely fresh medium and passaged weekly using Accutase (Stem Cell Technology). Upon splitting, the cells were cultured in mTESR1 medium supplement with 10 μM Y-27632 (Tocris) for 24 h and replaced with mTESR1 medium for further maintenance.

Adult HMVECs were purchased from Lonza (CC-2543) and cultured on an appropriate EC growth medium, which we refer to here as vessel medium (VM). The medium is composed of basal medium (CC-3156, Lonza) and supplements (CC-4147, Lonza), such as fetal bovine serum (FBS), hydrocortisone, human basic fibroblast growth factor (hFGF), VEGF, insulin-like growth factor (R3-IGF-1), ascorbic acid, human epidermal growth factor (hEGF), and gentamicin sulfate-amphotericin (GA-1000). The exact concentrations are undisclosed by the provider. The cells were expanded until passage 5 and used at that passage for 3D culture experiments with fibrin and for the angiogenesis assay on Matrigel.

Normal adult HDFs were purchased from Lonza (CC-2511) and cultured with fibroblast growth medium composed of basal medium (CC-3131) and supplements (CC-4126), such as FBS, insulin, hFGF, and GA-1000. Again, the exact concentrations are undisclosed by the provider. The cells were expanded until passage 6 and used for 3D culture experiments at that passage.

### SCs Isolation and Purification

Primary rat SCs were harvested from the sciatic nerves of neonatal Wistar rat pups, following local and Dutch animal use guidelines. Nerve segments were extracted and digested, followed by cell isolation and purification as described by Kaewkhaw et al.^76^ Briefly, the collected nerves were sliced and digested in a 0.05% (w/v) collagenase solution for 60 min at 37 °C, 5% CO_2_. The cell suspension was filtered through a 40 μm cell strainer, centrifuged for 6 min at 400*g*, followed by supernatant removal and cell pellet washing with Dulbecco’s modified Eagle’s medium (DMEM) containing 10% FBS, 100 U/mL penicillin and 100 μg/mL streptomycin. The cells were centrifuged again at 400*g* for 6 min, and the supernatant was discarded. Finally, the cells were resuspended with 2 mL of SC proliferation and purification medium, composed of DMEM d-valine (Cell Culture Technologies), 2 mM l-glutamine, 10% (v/v) FBS, 1% (v/v) N2 supplement (R&D Systems), 20 μg/mL bovine pituitary extract, 5 μM forskolin, 100 U/mL penicillin, 100 μg/mL streptomycin and 0.25 μg/mL amphotericin B (all Sigma-Aldrich), then plated on 35 mm Petri dish precoated with 0.01% (v/v) poly-l-lysine (Sigma-Aldrich) and 1 μg/mL laminin (R&D Systems) and incubated at 37 °C, 5% CO_2_. The use of d-valine in place of l-valine serves to inhibit fibroblast growth while permitting SCs survival and proliferation. Fresh medium (1 mL) was added on day 7 of culture and changed every 2 days until confluency. The cells were used between passage number 3 and 6 (P3–P6).

### iPSCs Differentiation and Neurosphere Formation

To induce iPSCs differentiation into nociceptors, we adapted and modified the protocol published by Chambers et al.^77^ Nociceptor induction was initiated using single-cell suspension of undifferentiated iPSCs detached with accutase, followed by seeding of 200 cells/microwell in mTESR1 medium supplemented with 10 μm Y-27632 and 0.5% Geltrex (in solution) onto 400 μm agarose microwells. The cell suspension was forced to settle by centrifugation at 290*g* for 2 min. Afterward, the cells were incubated for 24 h and were given a complete media change with mTESR1 medium. At this time, the cellular spheroid is formed and cell synchronization is initiated by the addition of mTESR1 medium supplemented with 1% dimethyl sulfoxide (DMSO). The cells were maintained for 72 h in the synchronization medium. Post-synchronization, the cells were given a PBS wash and nociceptor induction was initiated by the addition of dual SMAD inhibition media containing Advanced RPMI 1640 supplemented with Glutamax (both Thermo Fisher Scientific), 100 nM LDN-193189 (Tocris) and 10 μM SB431542 (Tocris). The spheres were maintained for 48 h in the dual SMAD inhibition media. Following this, neural crest commitment was induced via media containing advanced RPMI 1640 supplemented with Glutamax, 3 μM CHIR99021 (Tocris), and 1 μM retinoic acid (Tocris). The spheres were maintained in the neural crest induction media for 5 days with media change every alternate day. Following this stage, the spheres were incubated in Notch inhibition media, consisting of advanced RPMI supplemented with Glutamax, 10 μM SU5402 (Tocris), and 10 μM DAPT (Tocris) for 48 h.

Finally, the neurospheres, composed of trunk neural crest cells, were collected and seeded on coverslips or scaffolds. In these substrates, the cells were cultured in a neural maturation medium for at least 5 days to reach the nociceptor phenotype. The neural medium is composed of neurobasal medium, 0.5 mM Glutamax, 100 U/mL penicillin, 100 μg/mL streptomycin (all Thermo Fisher Scientific), 100 ng/mL human NGF, 50 μg/mL ascorbic acid (both Sigma-Aldrich), 25 ng/mL human neuregulin-1 type III (NRG-1 SMDF), and N21 supplement (both from R&D Systems).

### Scaffold Fabrication and Sterilization

The scaffolds were fabricated via a two-step electrospinning (ESP) process with a custom-built apparatus. The first step was the production of a release layer by electrospraying a solution of 50% poly(ethylene oxide) (PEO, Mn = 3350, Sigma-Aldrich) in Milli-Q onto aluminum foil. For this, the solution flowed through a 0.8 mm inner diameter, stainless steel needle (Unimed S.A.) at 2 mL/h, while subjected to 20 kV and at a distance of 10 cm from a 60 mm diameter mandrel rotating at 5000 rpm. Afterward, a nonwoven polyurethane mesh (6691 LL, 40 g/m^2^), a kind gift from Lantor B.V. (The Netherlands), was prepared by punching an array of 12 mm circular holes and placed on the mandrel, covering the PEO-sprayed foil. We then produced the scaffolds by ESP of 300PEOT55PBT45 (PolyVation) in 75:25 chloroform/1,1,3,3-hexafluoroisopropanol solution onto the mesh support structure. For this process, the solution flowed through a 0.5 mm inner diameter, stainless steel needle (Unimed S.A.) at 0.75 mL/h, while applying a voltage of 12 kV and at a distance of 10 cm from a rotating mandrel at 5000 rpm. During both processes, the humidity remained at 35–40% and the temperature at 22–24 °C. Finally, we generated individual scaffolds from the polyurethane mesh by punching 15 mm outer diameter sections concentric to the 12 mm holes, resulting in a thin ESP membrane supported by a polyurethane mesh ring. To detach the scaffolds, the mesh was dipped in deionized water and left in PBS until further use. When required for cell seeding, the scaffolds were transferred to a 24-well plate and immersed in 70% ethanol for 1 h for sterilization, followed by repeated PBS washes and air-drying. The scaffolds were maintained in sterile PBS until needed.

### Fabrication of a 3D PN Platform

To fabricate our PN platform, we followed the process illustrated in Figure 1A. While the iPSCs differentiated and formed neurospheres as described above, we simultaneously seeded the scaffolds with 100 × 10^3^ primary SCs and cultured these for 7 DIV with SC medium. During this time, the cells were allowed to populate the scaffold and align with its fibers to form highly anisotropic SCs bands. After 7 DIV, when SCs bands were fully formed, we added one neurosphere per scaffold. For this, we retrieved the neurospheres from the agarose mold into an Eppendorf tube, carefully pipetted one neurosphere onto each scaffold containing 125–150 μL of neural medium, and let the neurospheres adhere to the substrate for at least 6 h before adding neural medium. This medium was composed of the neurobasal medium, 0.5 mM Glutamax, 100 U/mL penicillin and 100 μg/mL streptomycin (all Thermo Fisher Scientific), 100 ng/mL human NGF, 50 μg/mL ascorbic acid (both Sigma-Aldrich), 25 ng/mL human neuregulin-1 type III (NRG-1 SMDF) and N21 supplement (both from R&D Systems). The following day, we added 300 μL of fibrin, composed of 3.5 mg/mL human fibrinogen (Enzyme Research Laboratories), 5 U/mL thrombin (Sigma-Aldrich), and 2.5 mM CaCl_2_. After full gel formation (∼15 min), neural medium containing 100 μg/mL aprotinin was added. The cultures were maintained for 7 or 21 DIV at 37 °C, 5% CO_2_, and the medium was refreshed every other day.

### Fabrication of a 3D Vascular Platform

The 3D vascularized platforms were fabricated through the co-culture of HMVECs with either HDFs or SCs, in a fibrin hydrogel. Briefly, the cells were counted and added to a tube at a density of 1.5 M cells/mL for HMVECs and 0.3 M cells/mL for HDFs or SCs. After this, the cells were resuspended in 150 μL of 10 mg/mL human fibrinogen plasminogen depleted (Enzyme Research Laboratories) and the suspension was added to either a 24-well plate or scaffold. Following this, 150 μL of thrombin at 20 U/mL and containing 5 mM CaCl_2_ was added to induce fibrin polymerization. The gels were allowed to form for approximately 15 min and then vessel medium (formulation above) was added. The samples were cultured for 10 DIV, with daily medium changes and at 37 °C, 5% CO_2_.

### Fabrication of the NV Platforms

To assemble the NV platforms, we followed two distinct strategies. The first strategy was used to assess the influence of different culture conditions, namely, cells and medium type, on the development of neural and vascular tissue. For this, we either coated the scaffold with laminin by adding 100 μL of 1 μg/mL laminin-1 (R&D Systems) and 2 μg/mL poly-d-lysine (Sigma-Aldrich) or seeded it with SCs (100 × 10^3^ cells/scaffold). The scaffolds were maintained in the SC proliferation medium for 7 days. After this, a neurosphere was placed at the center of both laminin and SC-seeded scaffolds, and the co-cultures were maintained in the neural medium for 7 DIV. On day 14, we added either 300 μL of blank fibrin or fibrin containing HMVECs (1.5 M/mL). The platforms were cultured for 10 DIV, in either the neural medium or a 1:1 mix of neural/vessel medium. For the second strategy, we aimed to build a mature NV platform by merging the previously formed vascular model and nerve model. The vascular model was composed of an HMVECs/SCs co-culture and formed as described in the above section. The vascular cultures were maintained for 11 DIV in the vessel medium. The nerve model was formed as described above, and the iPSCs/SCs co-cultures were maintained for 4 DIV in the neural medium. To make a single NV unit, we picked up the vascular model, which was formed over a polyurethane mesh ring that facilitated handling, and transferred it over the nerve model. Both components were bound together by dispersing 150 μL of fibrin over them and leaving it to polymerize for approximately 15 min. The cultures were supplemented with neural/vessel medium at 1:1 and kept for 10 DIV. All cultures were kept at 37 °C, 5% CO_2_, and the medium changed every other day.

### Collection of SCs or HDFs Conditioned Medium

SCs or HDFs were seeded at 10 × 10^3^ cells/cm^2^ on six-well plates in 1 mL of the normal vessel medium. The day after, the medium was removed and 2 mL of fresh medium was added. The cells were cultured for 5 DIV, and 1 mL of medium was collected every day and stored at −80 °C.

### Tube Formation Assay on Matrigel

For Matrigel coating, we dispensed 150 μL of ice-cold Matrigel (CB-40234A, Thermo Fisher Scientific) into the wells of a 48-well plate. The plate was incubated at room temperature (RT) for 10 min, followed by incubation at 37 °C for 30 min. After this, HMVECs were collected in the normal vessel medium or SC-conditioned vessel medium, and 250 μL of cell seeding solution was added into each well, at a density of 75 × 10^3^ cells/cm^2^. The cells were cultured for 48 h at 37 °C, 5% CO_2_.

### Cytokine Array

The detection of cytokines presence within normal vessel medium, SC-conditioned medium, and HDF-conditioned medium was performed with a cytokine array kit (AAR-CYT-2–2, Ray Biotech). The protocol was performed as indicated by the manufacturer, and the membranes were imaged with a chemiluminescence imaging system (Chemidoc, Bio-Rad) at an appropriate charge-coupled device (CCD) exposure.

### Immunostaining

Samples were fixed with 4% paraformaldehyde for 25 min at RT, rinsed thoroughly with PBS, and left in PBS until further use. Permeabilization and blocking were performed simultaneously with a solution of 1% Triton X-100, 5% goat serum, 0.05% Tween20, and 1% bovine serum albumin (BSA) in PBS, for 24 h at 4 °C, under mild agitation. The samples were then incubated for 48 h at 4 °C, under mild agitation, with primary antibody solutions containing 0.1% Triton X-100, 5% goat serum, 0.05% Tween20, and 1% BSA in PBS. The samples were then washed with a wash buffer composed of 0.05% Tween20 and 1% BSA in PBS, and left for 24 h at 4 °C, under mild agitation, to remove unbound antibodies. Secondary antibody solutions were prepared in wash buffer and incubated for 48 h at 4 °C, under mild agitation. Following this, we rinsed the samples with PBS, stained with DAPI (0.2 μg/mL) for 20 min at RT, and left them in PBS until imaging. For F-actin staining, we used Alexa Fluor 488- or Alexa Fluor 568-phalloidin (Thermo Fisher Scientific) at 1:75 dilution in PBS for 1 h at RT.

The used primary antibodies were: anti-βIII tubulin (Sigma-Aldrich, T8578, 1:500), anti-S100 (Sigma-Aldrich, S2644, 1:100), anti-myelin basic protein (MBP; Thermo Fisher, PA1-46447, 1:50), anti-CD31 (Agilent, M082329-2, 1:100), anti-CD31 (Abcam, ab32457, 1:100), anti-laminin (Bio-connect, LS-C384320, 1:100), anti-collagen IV (Nordic MUbio, MUB0338S, 1:100), anti-von Willebrand Factor (Abcam, ab194405, 1:100), anti-VE-cadherin (Cell Signalling Technology, D87F2, 1:100), and anti-α smooth muscle actin (Thermo Fisher Scientific, PA5–19465, 1:100).

The used secondary antibodies were goat anti-mouse conjugated with Alexa Fluor 488, goat anti-mouse conjugated with Alexa Fluor 568, goat anti-rabbit conjugated with Alexa Fluor 488, and goat anti-rabbit conjugated with Alexa Fluor 568 (all Thermo Fisher Scientific).

### Imaging

Bright-field images were acquired with an inverted microscope (Nikon Eclipse Ti-e). Fluorescent images were acquired with an inverted microscope (Nikon Eclipse Ti-e) or a confocal laser scanning microscope (Leica TCS SP8). For scanning electron microscopy (SEM), the samples were mounted on sample stubs with carbon tape and gold-sputtered for 40 s at 30 mA (Cressington Sputter Coater 108 auto), then imaged on a FEI/Philips XL-30 ESEM at *V* = 10 kV.

### Transmission Electron Microscopy (TEM) Imaging

Samples were prepared by fixation in 4% paraformaldehyde in PBS, followed by washing with 0.1 M cacodylate (3× for 15 min). The cells were fixed again with 2.5% glutaraldehyde in 0.1 M cacodylate overnight (minimum of 1 h), followed by washing with 0.1 M cacodylate (3× 15 min), postfixed with 1% osmiumteroxide + 1.5% potassium hexacyanoferrate(II) trihydrate in 0.1 M cacodylate, then washed again with 0.1 M cacodylate (3× for 15 min). Then, we proceeded to a dehydration series (70% for 30 min, 90% for 30 min, and 2× 100% for 30 min), followed by propylenoxide (2× 30 min) and propylenoxide:epon LX112 (1:1) overnight with stirring. The samples were covered with fresh epon LX112 for 7 h upon stirring and embedded in beemcapsules with fresh epon for 3 days at 60 °C. Sections of 60 nm were then cut with a diamond knife, stained with uranyl acetate and lead citrate, and imaged with the FEI Tecnai G2 Spirit BioTWIN iCorr TEM.

### Image Analysis

Two-dimensional images were processed and analyzed with Fiji software (https://fiji.sc/). Three-dimensional images and videos of neurons and vascular channels were processed with Amira (Thermo Fisher Scientific) or Leica Application Suit (LAS X, Leica Microsystems) software. To quantify neural morphological parameters, we captured images that contained the whole tissue sample. Neurite length was obtained with Simple Neurite Tracer plugin,^78^ by measuring the distance between cell bodies and the edge of the respective axons. Neurite area was obtained by first converting images of βIII tubulin^+^ cells to binary images and measuring the pixel area occupied by the neurites, excluding cell bodies. Then, we divided this value by the total area of the scaffold. To measure the orientation degree of HMVECs, we used the OrientationJ plugin^79^ and applied the Measure function over identical circular ROIs to obtain the coherence values (where 0 is full isotropy and 1 is full anisotropy). For this experiment, we took at least 10 measurements per sample. Vascular channel network analysis of 3D fibrin cultures was performed after acquisition of at least five images per sample. Vascular tube analysis of 2D Matrigel cultures was performed after acquiring images capturing the whole well where cells were cultured. Vessel/tube length was determined manually (by one user) by measuring the length of an individual vessel until the next bifurcation. Vessel/tube branching density was determined by manually counting the number of branching points within an image and dividing this value by the image area. Vessel/tube density was determined by manually counting the number of vessels within an image and dividing this value by the image area. Vessel area was obtained by converting images to binary and measuring the occupied area of CD31^+^ structures. This value was then divided by the total area of the image. Finally, to quantify the amount of detected cytokines in the membrane array images, we measured the integrated pixel density within identical circular ROIs manually positioned over the membrane spots. The final values were obtained by first correcting the sample’s signal through background subtraction. Then, we normalized the values on the conditioned medium samples by multiplying the spot value (individual cytokines) with the ratio between the positive control of the reference membrane (normal medium) and the sample membrane (conditioned media). This sample cytokine value was then divided by the corresponding reference cytokine value to obtain the final relative secretion value. Those values were then used to build a heat map, with different color codes according to the relative secretion value.

### Statistics

We analyzed the data and generated graphs using the software GraphPad Prism. Bar graphs are shown as mean ± SD, and boxplots represent data point between the minimal and maximal values. Statistical significances were determined employing an unpaired t-test or two-way analysis of variance (ANOVA) followed by a Tukey’s honestly significant difference (HSD) post hoc test (*p < 0.05, ** *p* < 0.01, ***p < 0.005, ****p < 0.0001 and ns is *p* > 0.05). The comparisons on the graphs from Figure 6 are relative to the laminin coating and blank fibrin samples (for each medium group).

## Supporting Information Available

SCs/HMVECs co-culture model validation, SC characterization, angiogenesis assay images, cytokine array membrane images, and vessel network quantification graphs (PDF)
Lumen formation in 3D vessels generated in SCs/HMVECs co-cultures (Movie S1) (MP4)
Lumen formation in aligned vessel networks from SCs/HMVECs co-cultures (Movie S2) (MP4)
SCs localization along the volume of the SCs/HMVECs aligned construct (Movie S3) MP4)

## Notes

Data underlying the figures can be found in the Supporting Data Source. All raw data are stored on https://datahub.io/ and are available from the authors upon reasonable request.
