# Supplementary material for: Development of an In Vitro Biomimetic Peripheral Neurovascular Platform
Source: ACS Appl Mater Interfaces. 2022 Jul 11;14(28):31567–85. doi: 10.1021/acsami.2c03861 (PMC9305708; doi:10.1021/acsami.2c03861)
Supplement: Supplementary file 4 — am2c03861_si_004.pdf [file am2c03861_si_004.pdf]

# Supporting information

## Development of an in vitro biomimetic peripheral neurovascular platform

Afonso Malheiro, Adrián Seijas Gamardo, Abhishek Harichandan, Carlos Mota, Paul Wieringa, Lorenzo Moroni*

Complex Tissue Regeneration Department, MERLN Institute for Technology-Inspired Regenerative Medicine, Maastricht University, 6229 ET Maastricht, Netherlands

Corresponding author: l.moroni@maastrichtuniversity.nl

**Supplementary Movie 1.** Lumen formation in 3D vessels generated in SCs/HMVECs co-cultures

**Supplementary Movie 2.** Lumen formation in aligned vessel networks from SCs/HMVECs co-cultures

**Supplementary Movie 3.** SCs localization along the volume of the SCs/HMVECs aligned construct

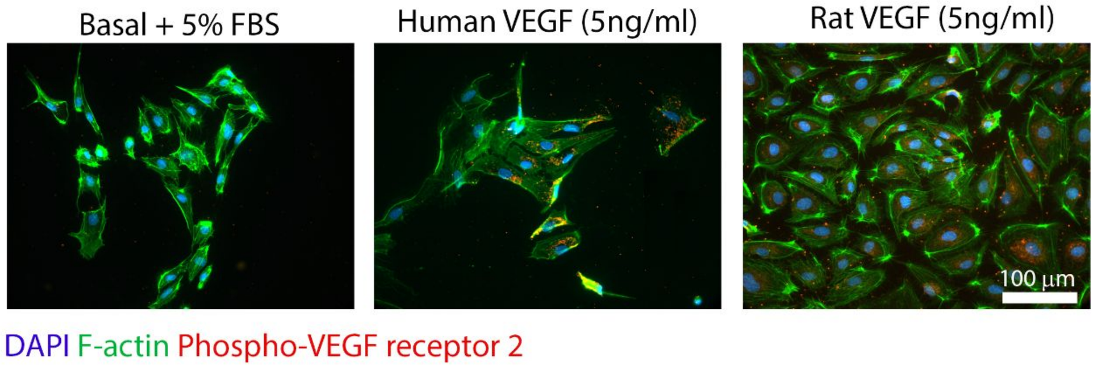


**Figure S1.** Both human and rat VEGF-165 can bind to the VEGF receptor-2 of HMVECs and induce its phosphorylation, as shown by phospho-VEGR2 immunostaining (in red). All samples were cultured in basal medium plus 5% FBS with either human VEGF (5ng/ml, middle image), rat VEGF (5ng/ml, right image) or no added VEGF (left image) for 24 h. F-actin is shown in green and DAPI in blue. Scale bar is 100 µm.

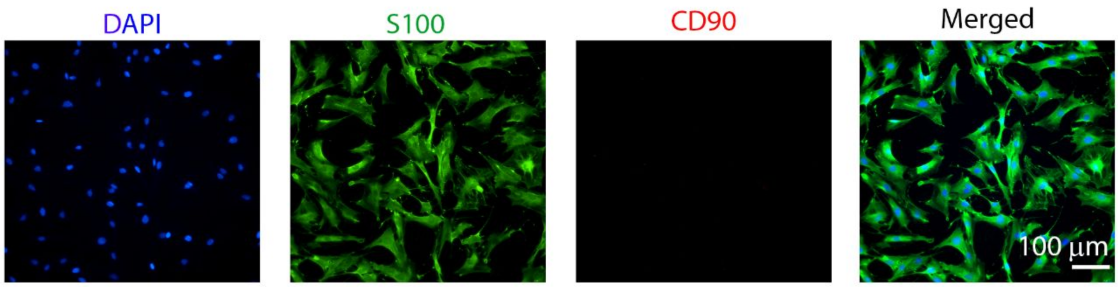


**Figure S2.** Characterization of the primary SCs isolated and purified from a rat sciatic nerve. All cells express S100 (green) and there were no signs of fibroblast contamination (CD90, red). DAPI is shown in blue and the scale bar is 100 µm.

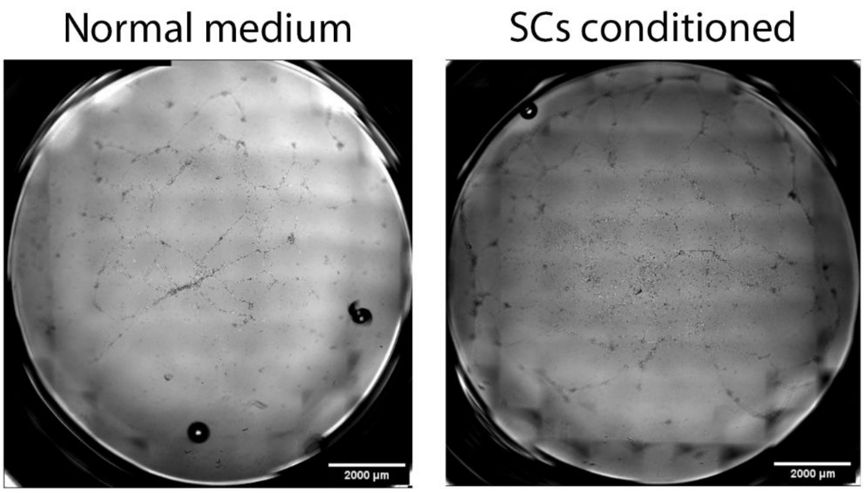


**Figure S3.** Brightfield micrograph showing tube formation from HMVECs, seeded onto matrigel-coated wells and cultured with normal vessel medium (left image) or SCs conditioned vessel medium (right image) for 48 h. Scale bar is 2000 µm.

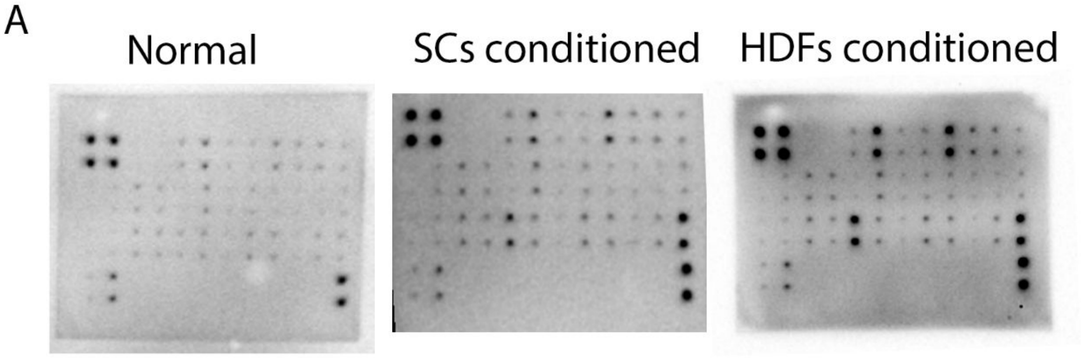


B

Each antibody is spotted in duplicate vertically

| | A | B | C | D | E | F | G | H | I | J | K | L |
|---|---|---|---|---|---|---|---|---|---|---|---|---|
| 1<br>2 | POS | POS | NEG | NEG | Activin A | Agrin | CD86 (B7-2) | beta-NGF | CINC-1 (CXCL1) | CINC-2 | CINC-3 | CNTF |
| 3<br>4 | Fas Ligand (TNFSF6) | Fractalkine (CX3CL1) | GM-CSF | ICAM-1 (CD54) | IFN-gamma | IL-1 alpha (IL-1 F1) | IL-1 beta (IL-1 F2) | IL-1 R6 (IL-1 Rrp2) | IL-2 | IL-4 | IL-6 | IL-10 |
| 5<br>6 | IL-13 | Leptin | LIX | L-Selectin (CD62L) | MCP-1 (CCL2) | MIP-3 alpha (CCL20) | MMP-8 | PDGF-AA | Prolactin R | RAGE | TCK-1 (CXCL7) | TIMP-1 |
| 7<br>8 | TNF alpha | VEGF-A | BLANK | BLANK | BLANK | BLANK | BLANK | BLANK | BLANK | BLANK | BLANK | POS |

**Figure S4.** A) Images of the cytokine array membrane after incubation with normal vessel medium (left image), SCs conditioned vessel medium (middle image) or HDFs conditioned vessel medium (right image). B) Manufacturer table of the detectable cytokines within the membrane. POS means positive control and NEG means negative control. BLANK refers to empty spots in the membrane.

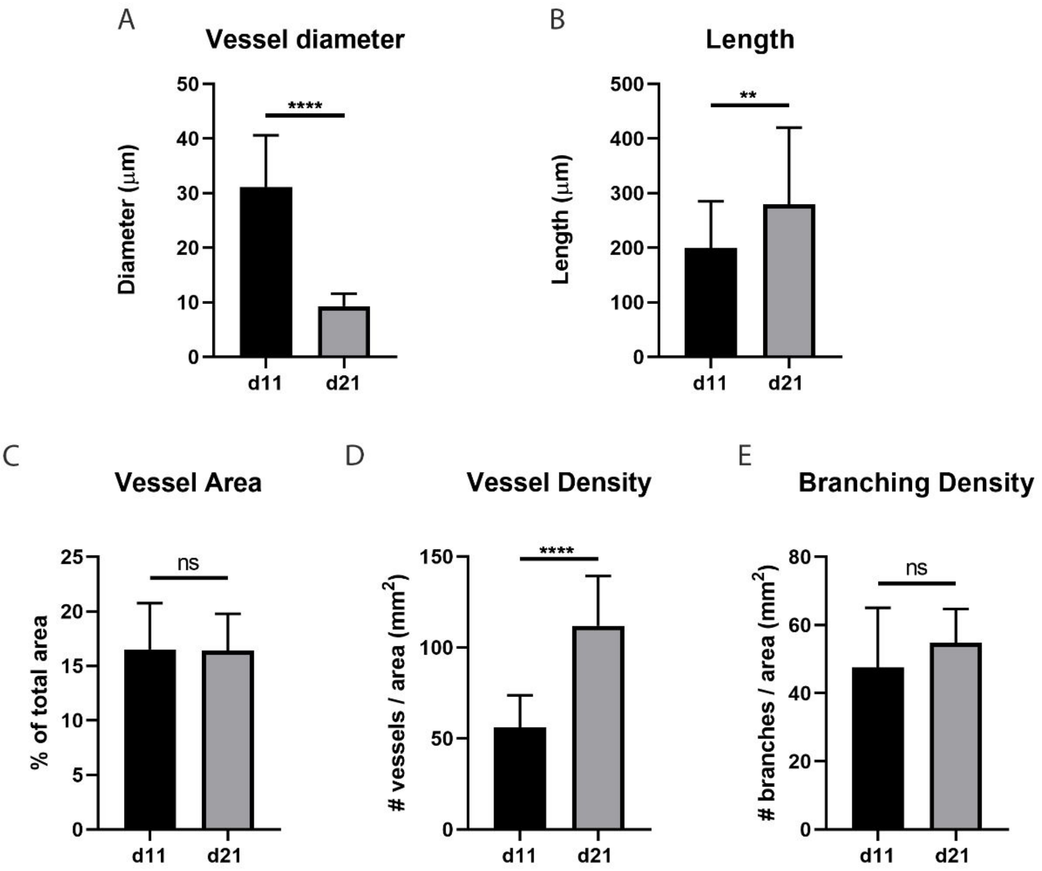


**Figure S5.** Characterization and comparison of formed vessel networks at 11 days (vasculature only platform) or 21 days of culture (neurovascular platform). 11 day cultures refer to the vessels in the vasculature only platform (prior to transfer to the neural platform), and are composed of solely HMVECs and SCs at 5:1, cultured in vessel medium. 21 days cultures refer to the vessels formed in the neurovascular platform. This is composed of 11 day vessel cultures that are transferred to a neural platform and cultured for additional 10 days in mixed medium (neural/vessel medium at 1:1), to form the neurovascular model. A) Vessel diameter, obtained from measuring the diameter of individual vessels. B) Vessel length, determined as the total length of an individual vessel until the next bifurcation. C) Vessel area was determined as the ratio of $CD31^+$ area per total image area. D) Vessel density, determined as the ratio of number of vessels contained in an image per image area. E) Branching density, determined as the ratio of vessel branching points contained in an image per image area. All graphs represent the mean ± SD. For the image analysis, we took at least five images per sample (n = 4). Statistics were performed using an unpaired t-test, where ****$p < 0.0001$, **$p < 0.01$ and ns denotes $p > 0.05$.
